# Preclinical evidence of probiotics in ulcerative colitis: a systematic review and network meta-analysis

**DOI:** 10.3389/fphar.2023.1187911

**Published:** 2023-06-09

**Authors:** Wenqin Jin, Huangping Ai, Qingqing Huang, Chuncai Li, Xiang He, Zhao Jin, Yuling Zuo

**Affiliations:** ^1^ Chengdu University of Traditional Chinese Medicine, Chengdu, China; ^2^ Hospital of Chengdu University of Traditional Chinese Medicine, Chengdu, China

**Keywords:** probiotics, ulcerative colitis, preclinical evidence, systematic review, network meta-analysis

## Abstract

The imbalance of gastrointestinal microbial composition has been identified as the main factor of chronic inflammatory diseases. At present, probiotics have a beneficial effect on the microbial composition of the human gastrointestinal tract, but it is still controversial and the specific mechanism is unknown. The purpose of this network meta-analysis is to compare the mechanism of different probiotics on ulcerative colitis. PubMed, Embase, and Web of Science were searched till 16 November 2022. The SYRCLE risk bias assessment tool was used to assess the quality of the research studies. A total of 42 studies, 839 ulcerative colitis models, and 24 kinds of probiotics were finally included. The results showed that *L. rhamnosus* has the best effect in relieving weight loss and improving the Shannon index in the ulcerative colitis model. *E. faecium* has the best effect in reducing colon injury; *L. reuteri* has the best effect in reducing the DAI; *L. acidophilus* has the best effect in reducing the HIS index and increasing the expression of tight junction protein ZO-1; and *L. coryniformis* has the best effect in reducing the content of serum pro-inflammatory factor TNF-α. It indicated that probiotics can improve ulcerative colitis by improving histopathological manifestations, reducing inflammatory reaction, and repairing the mucosal barrier, and different probiotics showed different effects. However, considering the limitations of this study, preclinical studies that require more large samples and high-quality and more reliable and rigorous experimental designs and reports need to be conducted in the future.

**Systematic Review Registration**: https://www.crd.york.ac.uk/prospero/#record details, identifier CRD42022383383.

## 1 Introduction

Ulcerative colitis (UC) is a chronic non-specific intestinal inflammatory disease characterized by inflammatory changes of colorectal mucosa. Its etiology is not clear, and it is difficult to cure and easy to become cancerous (Ungaro et al., 2017). Epidemiological research showed that the incidence of UC is rising all over the world (Charles et al., 2010). It is generally believed that the pathogenesis of UC is the result of the interaction of heredity, immunity, microorganism, and environment. At present, the traditional treatments for UC are mainly sulfasalazine, 5-aminosalicylic acid ester, glucocorticoid, and immunosuppressants (Travis et al., 2008; Kornbluth and Sachar, 2010), but their curative effects are limited, and there are many adverse reactions such as headache, nausea, and abdominal pain, and it is expensive that most patients cannot afford it. Therefore, it is a new trend to seek a safer and more effective adjuvant therapy or alternative therapy for UC.

Intestinal flora maintains the survival and metabolism of the body by ingesting nutrients in the body, which plays an important role in regulating the nutrition, metabolism, immunity, and pathogen defense of the host, and it is an important barrier to maintain the internal environment of the body. There are 100 trillion kinds of different microorganisms in the human intestine, including bacteria, fungi, viruses, and protozoa, which constitute the intestinal microflora (Zhang et al., 2020). Intestinal flora is considered to be one of the important organs symbiotic with the body, which is important to the occurrence and development of diseases. Intestinal flora imbalance can cause systemic inflammatory response. Probiotics are a vital component of intestinal flora, including *Bacteroides*, *Lactobacillus*, and *Bifidobacterium*. Supplementation of probiotics can improve the intestinal environment, including regulating the composition of intestinal flora, preventing colonization of pathogenic bacteria, affecting bacterial metabolism, and regulating immune response (Zhao et al., 2020).

There is significant imbalance of intestinal flora in UC patients, which is characterized by the decrease of the types and universality of intestinal microorganisms and the difference of their spatial distribution. A series of metabolites produced by intestinal flora also affect the occurrence and development of UC (Franzosa et al., 2019). A large number of animal and clinical studies have shown that probiotics can effectively alleviate the clinical symptoms and pathological damage of UC, improve the inflammatory reaction, and restore the balance of intestinal flora (Srutkova et al., 2015; Boruel et al., 2002), and have been widely used in the treatment of gastrointestinal diseases, but probiotics do not always provide benefits to patients, the effects of different strains are quite different, and the mechanism of action is also different, which is closely related to the type and stage of the disease, the strains and doses of probiotics used. However, at present, the functions and mechanisms of different probiotics have not been discussed. In this study, the effects of 24 probiotics on the DSS-induced UC mouse model were compared, and the advantages and disadvantages of different probiotics in the treatment of ulcerative colitis were summarized, which provided evidence for the selection of clinical probiotic strains.

## 2 Methods

The protocol of this network meta-analysis was registered on the International Prospective Register of Systematic Reviews (PROSPERO) (Registration ID: CRD 42022383383). The whole process is based on PRISMA guidelines (Moher et al., 2015).

### 2.1 Search strategy

We searched PubMed, Embase, and Web of Science for animal experiments on the treatment of ulcerative colitis with probiotics before 16 November 2022. The search keywords included “Probiotics,” “Ulcerative colitis,” and “Animals.” The search was limited to the English language. The specific search strategies can be obtained in Supplementary Table S1.

### 2.2 Eligibility criteria

Inclusion criteria: population (P): animal model of ulcerative colitis induced by DSS; intervention (I): the intervention of the experimental group was administration of a single probiotic, by gavage or intragastric, with no restrictions on the intervention dose and intervention period; comparison (C): the control group did not intervene or placebo intervened; outcomes (O): the main outcome indicators include weight change (WC), colon length (CL), disease activity index (DAI), and historical score (HIS).

Exclusion criteria: 1) vitro studies; 2) vivo studies with humans; 3) congresses, editorials, letters, case reports, and review works; 4) papers published in a language other than English; and 5) duplicate, abstracts, unpublished reports, or incomplete data and characteristic have been disregarded.

### 2.3 Data extraction and quality assessment

The two authors screened the literature according to the inclusion and exclusion standard independently, and then, they extracted the following information: author, publication year, animal species (sex, n, weight), UC model induction method, study design (probiotics, administration, dose, and duration), and outcome indicators. If there were different doses of the same probiotic, the dose with the best effect was selected. When there was only SEM but no SD in the text, we converted it by the formula SD = SEM*√n (Lee et al., 2015). We used WebPlotDigitizer 4.5 software (https://automeris.io/WebPlotDigitizer) to quantify the data of that only have pictures in the text. We had tried to contact the original author when the data were unavailable. We used the SYRCLE Risk Bias Tool to evaluate the risk of bias (Hooijmans et al., 2014), including sequence generation, baseline characteristics, allocation concealment, random housing, blinding of outcome assessors, random outcome assessment, blinding of experimentalist, incomplete outcome data, selective outcome reporting, and other sources of bias, every aspect classified as high risk of bias and low risk of bias or unclear. In case of any doubt in the aforementioned process, a third author was involved.

### 2.4 Outcomes

The primary outcomes were WC, CL, DAI, and HIS. The secondary outcomes were tumor necrosis factor-α (TNF-α), zonula occlusion ns-1 (ZO-1), and Shannon.

### 2.5 Data synthesis and statistical analysis

We used R 4.2.2 to analyze the data, and Stata16 was used to make a network evidence diagram and comparison-correction funnel diagram. Mean difference (MD) and 95% credible intervals (CrIs) were used to assess continuous outcomes, except TNF-α which used standard mean difference (SMD) and 95% credible intervals (CrIs). The network meta-analysis was carried out based on the Markov chain-Monte Carlo method in the Bayesian framework, and 4 Markov chains, 1 thinning interval, 20,000 burn-in, and 50,000 iterations were set. The convergence degree of the model is satisfactory when the potential scale reduction factor (PSRF) tends to 1(Supplementary Figure S1). When there was a closed loop, the node analysis model was used to test the inconsistency.

## 3 Results

### 3.1 Study selection

**FIGURE 1:**
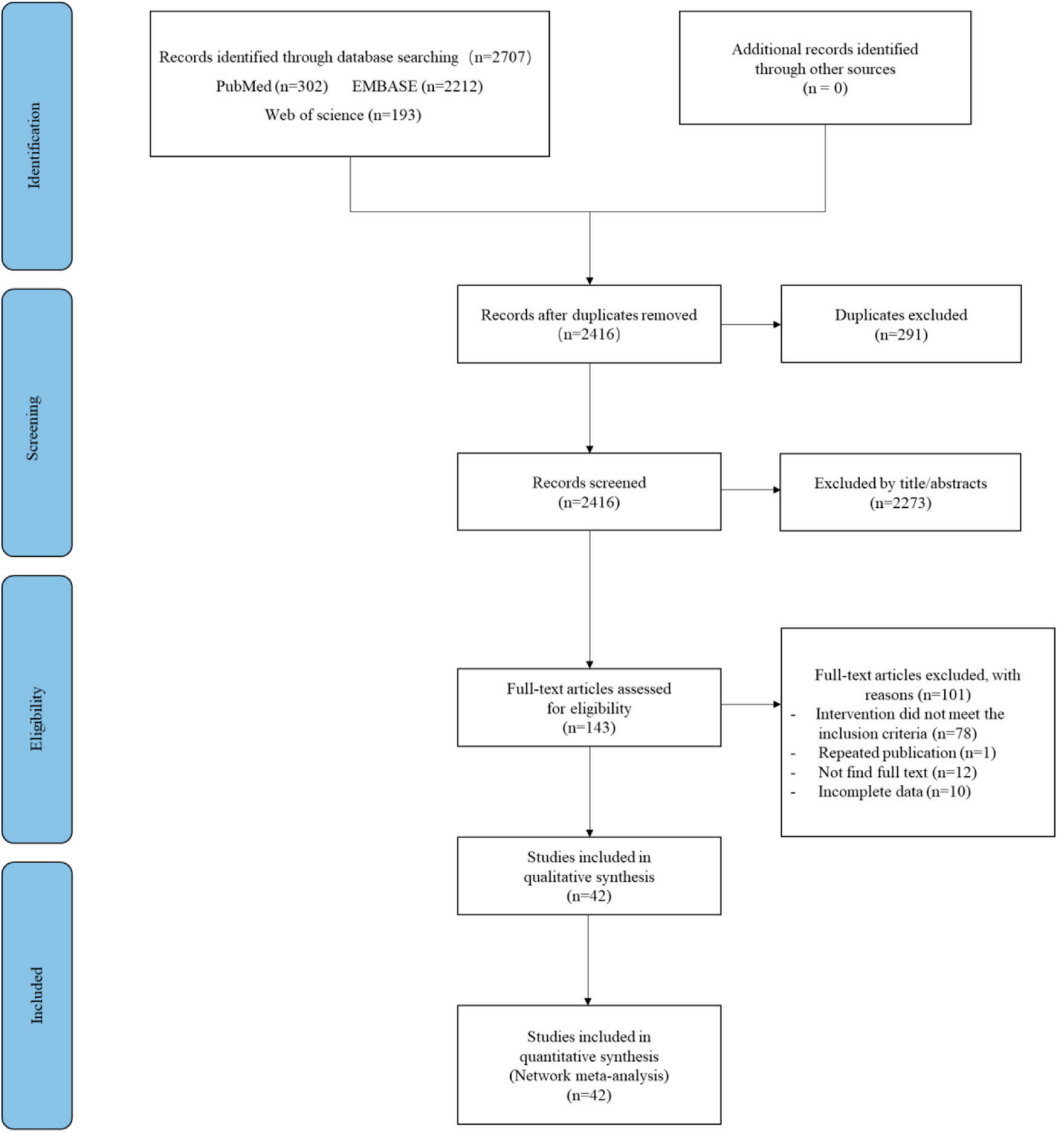
PRISMA flow chart for the study selection process.

A total of 2,707 studies were obtained, 291 studies duplicates were removed, 2,273 studies were excluded after reading the title/abstract, and 65 studies were retained after reading the full text according to the PICO inclusion criteria, and then, through further screening, 1 study was repeatedly published, we were unable to obtain the full text from 12 studies, and 10 studies had incomplete data. Finally, 42 studies were included in the network meta-analysis (Liu et al., 2011; Zakostelska et al., 2011; Chen et al., 2012; Chen et al., 2013; Pan et al., 2014; Elian et al., 2015; Lee et al., 2015; Cui et al., 2016; Kanda et al., 2016; Liu et al., 2016; Chae et al., 2018; Sun et al., 2018; Wang et al., 2018; Bian et al., 2019; Chae et al., 2019; Chen et al., 2019; Choi et al., 2019; Din et al., 2020; He et al., 2020; Hu et al., 2020; Sun et al., 2020; Yeo et al., 2020; Yu et al., 2020; Chen et al., 2021; Gao et al., 2021; Hu et al., 2021; Huang et al., 2021; Qu et al., 2021; Tong et al., 2021; Wang et al., 2021; Dong et al., 2022; Huang et al., 2022; Islam et al., 2022; Khan et al., 2022; Lee et al., 2022; Li et al., 2022; Lu et al., 2022; Ma et al., 2022; Qin et al., 2022; Shang et al., 2022; Wan et al., 2022; Wu et al., 2022). The specific selection flow chart was shown in Figure 1.

### 3.2 Study characteristics

A total of 42 articles were included, and the publication time was concentrated between 2011 and 2022. There were 839 ulcerative colitis animal models, including 460 in the intervention group and 379 in the control group. There were four different kinds of mice, and 14 studies used the BALB/c mice (Lee et al., 2022; Chen et al., 2013; Liu et al., 2016; Pan et al., 2014; Liu et al., 2011; Kanda et al., 2016; Cui et al., 2016; Chen et al., 2019; Zakostelska et al., 2011; Elian et al., 2015; Sun et al., 2018; Yu et al., 2020; Wang et al., 2018; Lee et al., 2015), with a total of 306 mice, 24 studies used the C57BL/6 mice (Chen et al., 2012; Chae et al., 2018; Bian et al., 2019; Chae et al., 2019; Din et al., 2020; He et al., 2020; Hu et al., 2020; Yeo et al., 2020; Chen et al., 2021; Gao et al., 2021; Hu et al., 2021; Huang et al., 2021; Qu et al., 2021; Tong et al., 2021; Dong et al., 2022; Huang et al., 2022; Islam et al., 2022; Khan et al., 2022; Lu et al., 2022; Ma et al., 2022; Qin et al., 2022; Shang et al., 2022; Wan et al., 2022; Wu et al., 2022), with a total of 436 mice, 2 studies used the ICR mice (Sun et al., 2018; Choi et al., 2019), with a total of 32 mice, 1 study used the Kunming mice (Wang et al., 2021), with 35 mice in total, and 1 study used the Sprague Dawley (SD) mice (Li et al., 2022), with a total of 20 mice. All studies adopted animal models of ulcerative colitis induced by dextran sulfate sodium (DSS), and the administration duration ranged from 7 days to 35 days. There were 24 kinds of probiotics in total, and seven studies used *Bifidobacterium* (*B. bifidum*) (Elian et al., 2015; Chae et al., 2018; Wang et al., 2018; Din et al., 2020; Dong et al., 2022; Lee et al., 2022; Shang et al., 2022), thirteen studies used *Lactobacillus plantarum* (*L. plantarum*) (Liu et al., 2011; Lee et al., 2015; Cui et al., 2016; Chae et al., 2019; Choi et al., 2019; Sun et al., 2020; Yu et al., 2020; Hu et al., 2021; Huang et al., 2021; Wang et al., 2021; Khan et al., 2022; Qin et al., 2022; Wu et al., 2022), two studies used Akkermansia muciniphila (AKK) (Bian et al., 2019; Qu et al., 2021), two studies used *Lactobacillus fermentum* (*L. fermentum*) (Cui et al., 2016; Chen et al., 2021), four studies used *Lactobacillus rhamnosus* (*L. rhamnosus*) (Yeo et al., 2020; Tong et al., 2021; Wang et al., 2021; Wan et al., 2022), three studies used *Lactobacillus bulgaricus* (*L. bulgaricus*) (Hu et al., 2020; Chen et al., 2021; Hu et al., 2021), and two studies used *Lactobacillus paracasei* (*L. paracasei*) (Pan et al., 2014; Huang et al., 2021). There was one study using *Saccharomyces boulardii* (*S. boulardii*) (Gao et al., 2021), *Clostridium butyricum* (*C. butyricum*) (Ma et al., 2022), *Lactobacillus brevis* (*L. brevis*) (Liu et al., 2016), *Lactobacillus crispatus* (*L. crispatus*) (Cui et al., 2016), *Lactobacillus reuteri* (*L. reuteri*) (Sun et al., 2018), *Lactobacillus neoformans* Lb-9 (LB-9) (Chae et al., 2019), *Lactobacillus casei* (*L. casei*) (Zakostelska et al., 2011), *Lactobacillus kefiranofaciens* M1 (*L. kefiranofaciens* M1) (Chen et al., 2012), *Eurotium cristatum* (*E. cristatum*) (Lu et al., 2022), *Lactobacillus acidophilus* (*L. acidophilus*) (Hu et al., 2020), *Enterococcus durans* (*E. durans*) (Kanda et al., 2016), *Streptococcus thermophilus* (*S. thermophilus*) (Chen et al., 2019), *Tetragenococcus halophilus* (*T. halophilus*) (Islam et al., 2022), *Enterococcus faecalis* (*E. faecium*) (Wang et al., 2021), *Lactobacillus crustorum* (*L. crustorum*), and *Lactobacillus coryniformis* (*L. coryniformis*) (Wang et al., 2021). A total of seven outcome indicators were analyzed, including WC, Cl, DAI, HIS, TNF-α, ZO-1, and Shannon. The specific information is presented in Table 1.

**TABLE 1:** Basic characteristics of the included study.

Study (year)	Species (sex, n = C/E, weight)	UC model induction method	Control (administration, drug, dose)	Experiment (administration, drug, dose)	Duration	Outcomes
Lee et al. (2022)	BALB/c mice	2.5% DSS, 5 days	Gavage, PBS 200 μL	Gavage, *B. bifidum*	15 days	①②④
	(female, 12/12)			10^9^ CFU/mouse/day		
Chen et al. (2013)	BALB/c mice (female, 8/8, 20.0 ± 2.0 g)	5% DSS, 7 days	Gavage, normal saline	Gavage, *L. acidophilus*, 10^6^ CFU/10 g	7 days	②③④
Gao et al. (2021)	C57BL/6 mice (female, 20/20, 20–24 g)	2.5% DSS, 7 days	Gavage, distilled water	Gavage, *S. boulardii*, 10^7^ CFU/mL	28 days	①
Ma et al. (2022)	C57BL/6 mice (male, 8/8)	3% DSS, 7 days	Gavage, normal saline	Gavage, *C. butyricum*, 10^8^ CFU	28 days	①②③④⑤⑥
Liu et al. (2016)	BALB/c mice (female, 8/8)	5% DSS, 7 days	Gavage, 0.2 mL PBS	Gavage, *L. brevis*, 10^9^ CFU	14 days	①②
Chen et al. (2021)	C57BL/6J mice (male, 10/10/10)	3% DSS, 14 days	Gavage, normal saline	Gavage	35 days	②⑤
				*L. fermentum*, *L. bulgaricus*		
				10^9^ CFU/kg		
Huang et al. (2021)	C57BL/6 mice (6/6)	3% DSS, 7 days	Gavage, distilled water	Gavage, *L. paracasei*, 10^9^ CFU/mL	14 days	②
Li et al. (2022)	Sprague Dawley rats (male, 10/10)	5% DSS, 8 days	Intragastric, sterilized water	Intragastric	7 days	③④⑤
				*L. acidophilus*, 10^8^ CFU		
Chae et al. (2018)	C57BL/6 mice	3% DSS, 6 days	Gavage, drinking water	Gavage, *B. bifidum*, 1.2 × 10^10^ CFU	7 days	②③
	(male, 7/7)					
Pan et al. (2014)	BALB/c mice (male, 10/10, 22.0 ± 2.0 g)	2.5% DSS, 7 days	Gavage, normal saline	Gavage, *L. paracasei*, 10^10^ CFU/mL	14 days	②③④
Liu et al. (2011)	BALB/c mice (female,8/8)	5% DSS, 7 days	Gavage, 0.2 mL PBS	Gavage, *L. plantarum*, 10^9^ CFU in 0.2 mL PBS	14 days	①②③④
Din et al. (2020)	C57BL/6 mice (male, 7/7)	3% DSS, 7 days	Gavage, PBS	Gavage, *B. bifidum*, 10^9^ CFU/mL	27 days	②③④⑤⑥
Shang et al. (2022)	C57BL/6 mice (male, 8/8, 22 ± 2.0 g)	2.5% DSS, 7 days	Gavage, normal saline	Gavage, *B. bifidum*, 10^9^ CFU/d	14 days	②
Sun et al. (2018)	ICR mice (female, 8/8, 24–28 g)	3.5% DSS, 7 days	Gavage, distilled water	Gavage	17 days	②③④
				*L. reuteri*, 10^11^ CFU/mL		
Qin et al. (2022)	C57BL/6 mice (male, 10/10)	3% DSS, 7 days	Gavage, normal saline	Gavage, *L. plantarum*	7 days	②③④
				5 × 10^8^ CFU/10 g		
Kanda et al. (2016)	BALB/c mice (females, 10/10)	4% DSS, 5 days	Gavage, PBS	Gavage, *E. durans*, 10 mg/day in 0.3 mL PBS	12 days	③④⑦
Qu et al. (2021)	C57BL/6 mice (male, 5/5)	3% DSS, 8 days	Gavage, 300 mL PBS	Gavage, AKK, 10^9^ CFU in 300 mL PBS	15 days	④
Wang et al. (2021)	Kunming mice (male, 7/7/7/7/7)	2% DSS, 7 days	Gavage, 200 μL PBS	Gavage, *L. rhamnosus*	14 days	⑤⑥
				*L. plantarum*, *L. crustorum*		
				*L. coryniformis*, 5 × 10^9^ CFU/mL		
Cui et al. (2016)	BALB/c mice (female,20.0 ± 2.0g, 10/10/10/10)	5% DSS, 7 days	Gavage, drink water	Gavage, *L. fermentum*, *L. crispatus*, *L. plantarum*, 0.4 mL of 3.0 × 10^8^ CFU/mL	9 days	①②③
Bian et al. (2019)	C57BL/6 mice (male, 8/8)	2% DSS, 7 days	Gavage, PBS	Gavage, AKK, 3 × 10^9^ CFU	14 days	①②③④⑤
He et al. (2020)	C57BL/6 mice (Male, 16/16, 22–24 g)	3% DSS, 7 days	Gavage, PBS	Gavage, *E. faecium*, 10^9^ CFU/day	14 days	①②⑤
Chen et al. (2019)	BALB/c mice (male, 10/10)	2.5% DSS, 7 days	Gavage, distilled water	Gavage, *E. durans*, 200 mg/kg	14 days	②③④
Hu et al. (2020)	C57BL/6 mice (male, 10/10/10)	3% DSS, 7 days	Intragastric, normal saline	Intragastric, *L. acidophilus*	21 days	②⑤⑥
				*L. bulgaricus*, 10^9^ CFU/mL		
Islam et al. (2022)	C57BL/6 mice (4/4)	4% DSS, 7 days	Gavage, drink water	Gavage, *T. halophilus*, 4.8 × 10^8^ CFU/mouse/day	10 days	②③⑦
Tong et al. (2021)	C57BL/6J mice (male, 7/7)	3.5% DSS, 7 days	Gavage, drinking water	Gavage, *L. rhamnosus*, 1.2 mg/kg	14 days	①②⑦
Yeo et al. (2020)	C57BL/6J mice (female, 4/4)	1.5% DSS, 6 days	Gavage, PBS	Gavage, *L. rhamnosus*, 10^9^ CFU/day	26 days	②⑥
Wan et al. (2022)	C57BL/6 mice (female, 10/10)	2.5% DSS, 7 days	Gavage, drinking water	Gavage, *L. rhamnosus*, 600 mg kg^−1^ day^−1^	15 days	②③
Dong et al. (2022)	C57BL/6 mice (male, 10/10)	2.5% DSS, 7 days	Gavage, normal saline	Gavage, *B. bifidum*, 10^9^ CFU	12 days	①③
Chae et al. (2019)	C57BL/6 mice	3% DSS, 6 days	Gavage, drinking water	Gavage, LB-9, 1.2 × 10^10^ CFU	7 days	②③
	(male, 7/7)					
Zakostelska et al. (2011)	BALB/c mice (female, 5/5)	3% DSS, 7 days	No mention	Gavage, *L. casei*, 1.5 mg of Lc in 50 mL of sterile PBS	21 days	②③④
Elian et al. (2015)	BALB/c mice (female, 12/12)	3.5% DSS, 7 days	Gavage, 0.1 mL PBS	Gavage, *B. bifidum*, 0.1 mL	10 days	③
Sun et al. (2020)	BALB/c mice (male, 15/15, 18 g–20 g)	5% DSS, 7 days	Gavage, normal saline	Gavage, *L. plantarum*, 10^9^ CFU/mL	10 days	①②⑤⑦
Choi et al. (2019)	ICR mice (male, 8/8)	1.5% DSS	Gavage, 0.2 mL of PBS	Gavage, *L. plantarum*, 0.2 mL	21 days	②
Huang et al. (2021)	C57BL/6 mice (male, 8/8)	4% DSS, 7 days	Gavage, distilled water	Gavage, *L. plantarum*, 0.2 mL/10 g	7 days	②③⑤⑦
Wu et al. (2022)	C57BL/6 mice (male, 6/6, 20–22 g)	3.5% DSS, 7 days	Gavage, 200 μL PBS	Gavage, *L. plantarum*, 200 μL 10^9^ CFU mL^−1^	7 days	①②③
Yu et al. (2020)	BALB/c mice (10/10)	3.5% DSS, 7 days	Gavage, normal saline	Gavage, *L. plantarum*, 10^10^ CFU/mL	28 days	②③
Hu et al. (2020)	C57BL/6 mice (male, 10/10/10, 25 ± 2 g)	2% and 4% DSS, weeks 3 and 5	Gavage, drinking water	Gavage, *L. plantarum*, *L. bulgaricus*, 0.2 mL of 10^9^ CFU/mL	35 days	②③
Wang et al. (2018)	BALB/c mice (female, 10/10/10, 16–18 g)	3% DSS, 7 days	Gavage, drinking water	Gavage, *L. plantarum*, *B. bifidum*, 300 µL 10^9^ CFU/mL	7 days	③
Khan et al. (2022)	C57BL/6 mice (female, 4/4, 18 ± 2 g)	2.5% DSS, 7 days	Intragastric, distilled water	Intragastric, *L. plantarum*, 10^10^ CFU/mL	21 days	①②④⑦
Chen et al. (2012)	C57BL/6 mice (female, 8/8)	2% DSS, 7 days	Intragastric, PBS	Intragastric, *L. kefiranofaciens* M1, 10^8^ CFU	14 days	②
Lu et al. (2022)	C57BL/6 mice	1.5% DSS, 7 days	Gavage, normal saline	Gavage, *E. cristatum*, 50 mg/mL	7 days	②③
	(male, 10/10)					
Lee and Lee (2015)	BALB/c mice (male, 10/10, 18–20 g)	2% DSS, 7 days	Gavage, 0.2 mL PBS	Gavage, *L. plantarum* 2.0 × 10^11^ CFU/kg	14 days	①②⑤

①WC, ②CL, ③DAI, ④HIS, ⑤TNF-α, ⑥ZO-1, ⑦Shannon.

### 3.3 Risk of bias assessment

**FIGURE 2:**
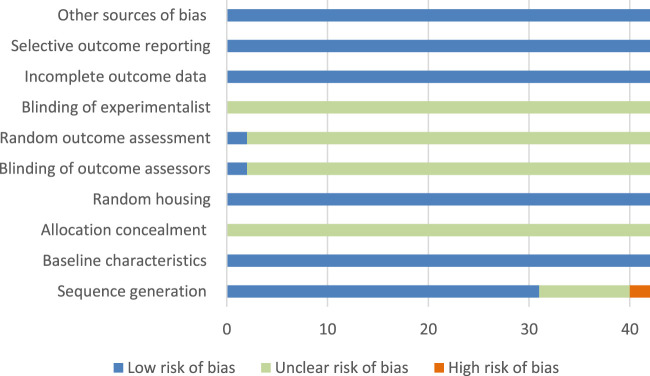
Risk of bias graph and evaluators; none of the studies mentioned the use of blind method; no research reported incomplete data, selective report bias, and other bias, so they were considered as low risk.

The ROB assessment processes and details of 10 projects in 42 studies are shown in Supplementary Table S2 and Figure 2. Randomization was used in 31 studies, and nine studies did not mention the use of random method, and two studies did not use randomization; all studies did not mention the hidden distribution sequence, so we considered it was unclear risk; all studies mentioned Random housing, so it was considered as low risk; and two studies mentioned the blind use of outcome assessment.

### 3.4 Outcomes

The primary outcomes were WC, CL, DAI, and HIS. The secondary outcomes were TNF-α, ZO-1, and Shannon.

#### 3.4.1 Weight change

**FIGURE 3:**
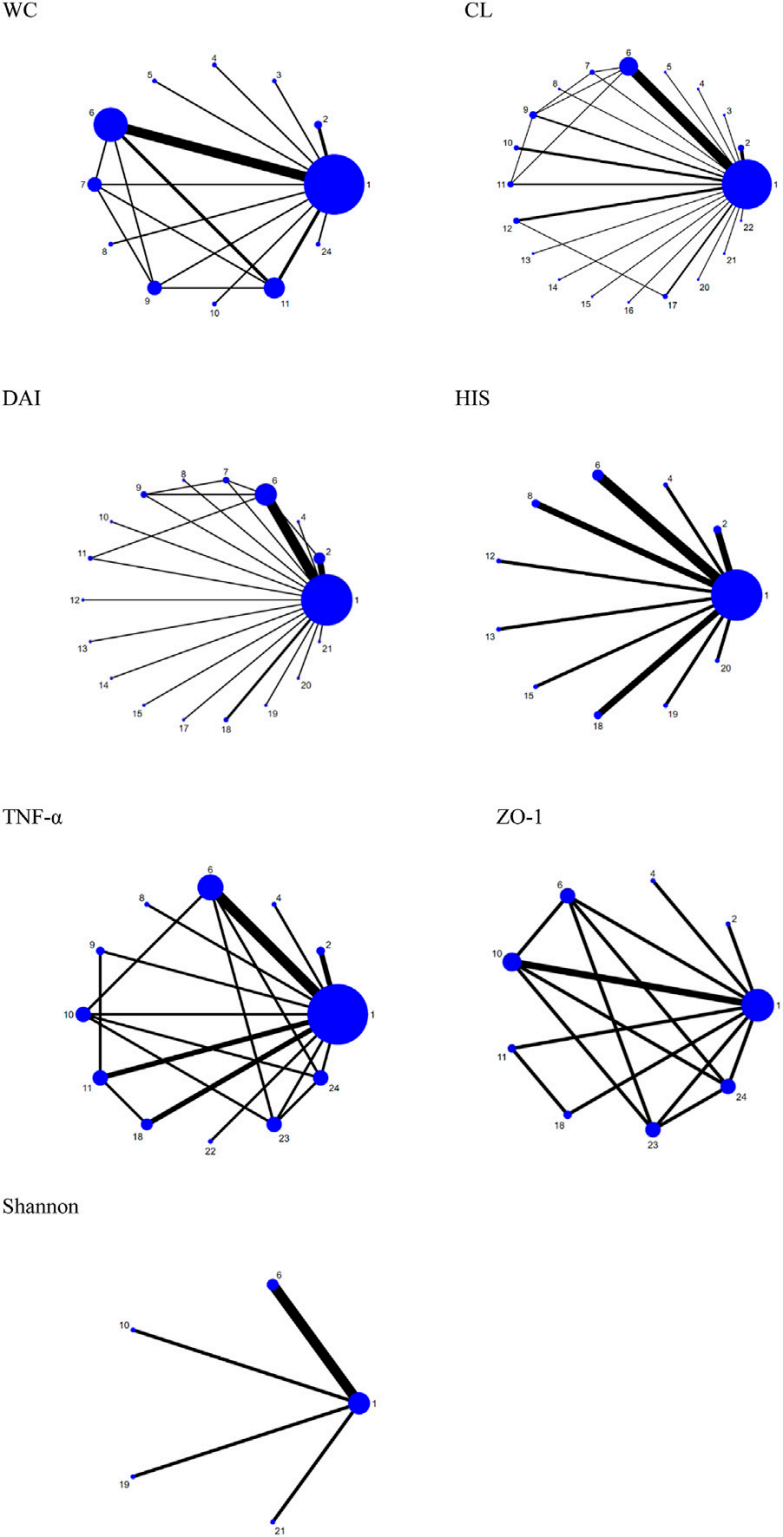
Network plots for different outcomes. 1. DSS; 2. *B. bifidum*; 3. *S. boulardii*; 4. *C. butyricum*; 5. *L. brevis*; 6. *L. plantarum*; 7. *L. crispatus*; 8. AKK; 9. *L. fermentum*; 10. *L. rhamnosus*; 11. *L. bulgaricus*; 12. *L. paracasei*; 13. *L. reuteri*; 14. LB-9; 15. *L. casei*; 16. *L. kefiranofaciens* M1; 17. *E. cristatum*; 18. *L. acidophilus*; 19. *E. durans*; 20. *S. thermophilus*; 21. *T. halophilus*; 22. *E. faecium*; 23. *L. crustorum*; and 24. *L. coryniformis*.

**FIGURE 4:**
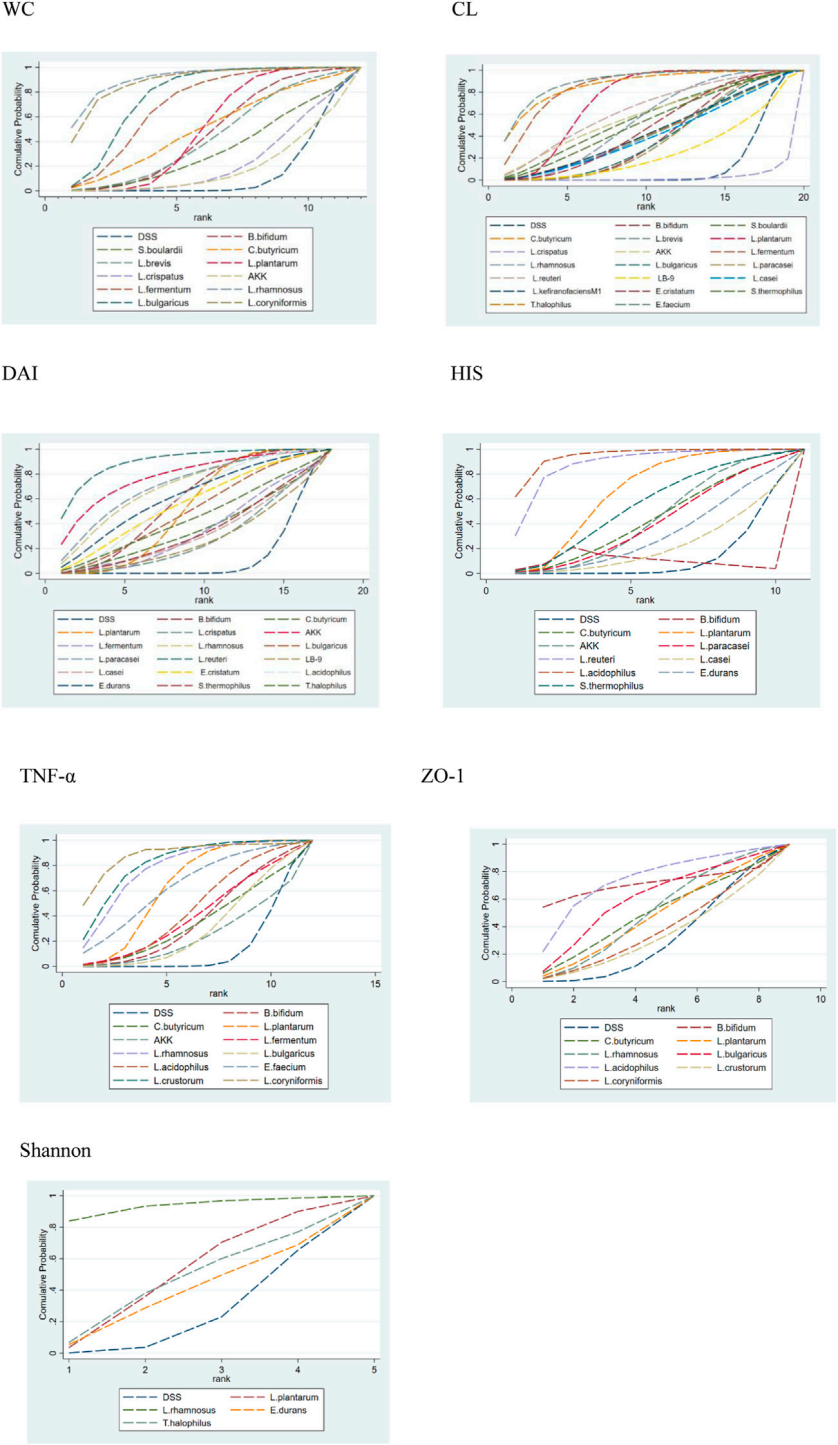
Surface under the cumulative ranking curve (SUCRA) plots for different outcomes. The vertical axis represents cumulative probabilities and the horizontal axis represents rank. 1. WC, 2. CL, 3. DAI, 4. HIS, 5. TNF-α, 6. ZO-1, and 7. Shannon.

Fourteen research studies reported the outcome indicators of WC, a total of 304 UC models, involving 11 probiotics. The network evidence diagram is shown in Figure 3. Compared with the DSS group, the weight loss of 11 probiotics decreased, among which *L. rhamnosus* (MD = 2.87, 95% CrI (1.02, 4.73)), *L. bulgaricus* (MD = 1.89, 95% CrI (0.88, 2.88)), *L. coryniformis* (MD = 2.68, 95% CrI (0.88, 4.48)), *L. plantarum* (MD = 1.03,95% CrI (0.41, 1.70)), and *L. fermentum* (MD = 1.67,95% CrI (0.34, 2.97)) had statistical significance. Meanwhile, *L. rhamnosus* (MD = −2.64,95% CrI (−4.94, −0.38)) (MD = −2.82,95% CrI (−5.18, −0.46)), *L. bulgaricus* (MD = −1.66, 95% CrI (−3.04, −0.28)) (MD = −1.84, 95% CrI (−3.60, −0.05)), and *L. coryniformis* (MD = −2.45, 95% CrI (−4.70, −0.23)) (MD = −2.63, 95% CrI (−4.95, −0.33)) were obviously superior to *L. crispatus* and AKK in weight reduction, and there was no statistical difference compared with other probiotics. The specific data are shown in Table 2. According to the SUCRA probability results (Figure 4), *L. rhamnosus* (91.05%) > *L. coryniformis* (88.66%) > *L. bulgaricus* (77%) > *L. fermentum* (9.80%) > *L. plantarum* (49.73%) > *C. butyricum* (49.91%) > L. brevis (43.14%) > *S. boulardii* (32.44%) > *L. crispatus* (21.99%) > AKK (17.57%) > DSS (12.36%), suggesting that *L. rhamnosus* was the best strain to reduce the weight loss of UC.

**TABLE 2:** League table for WC; results of the network meta-analysis are presented in the left lower half and results from pairwise meta-analysis in the upper right half. In the left lower and upper right half, mean differences lower than 0 favor the column-defining treatment, and bold font indicates significant results. NA: not available.

DSS	−2.9 (−4.6, −1.2)	−1.8 (−2.9, −0.85)	−0.95 (−1.9, 0.045)	−2.7 (−4.3, −1.0)	−0.50 (−2.1, 1.1)	−1.1 (−2.8, 0.69)	−0.85 (−2.1, 0.45)	−1.1 (−1.7, −0.51)	0.42 (−1.3, 2.1)	−0.051 (−1.3, 1.2)	−1.0 (−2.6, 0.52)
**2.87 (1.02, 4.73)**	***L. rhamnosus***	NA	NA	NA	NA	NA	NA	NA	NA	NA	NA
**1.89 (0.88, 2.88)**	−0.98 (−3.10, 1.12)	***L. bulgaricus***	NA	NA	NA	NA	NA	0.96 (−0.17, 1.9)	**1.5 (0.20, 2.9)**	NA	0.11 (−1.2, 1.4)
0.94 (−0.18, 2.045)	−1.93 (−4.11, 0.22)	−0.95 (−2.45, 0.56)	***B. bifidum***	NA	NA	NA	NA	NA	NA	NA	NA
**2.68 (0.88, 4.48)**	−0.19 (−2.76, 2.40)	0.79 (−1.25, 2.87)	1.74 (−0.36, 3.87)	***L. coryniformis***	NA	NA	NA	NA	NA	NA	NA
0.50 (−1.29, 2.28)	−2.37 (−4.94, 0.21)	−1.39 (−3.42, 0.65)	−0.44 (−2.53, 1.66)	−2.17 (−4.73, 0.35)	***S. boulardii***	NA	NA	NA	NA	NA	NA
1.07 (−0.83, 2.96)	−1.81 (−4.48, 0.86)	−0.82 (−2.97, 1.34)	0.12 (−2.07, 2.33)	−1.62 (−4.23, 1.00)	0.56 (−2.03, 3.17)	***C. butyricum***	NA	NA	NA	NA	NA
0.85 (−0.64, 2.35)	−2.02 (−4.40, 0.36)	−1.04 (−2.81, 0.76)	−0.09 (−1.93, 1.79)	−1.83 (−4.17, 0.51)	0.35 (−1.97, 2.69)	−0.21 (−2.60, 2.19)	***L. brevis***	NA	NA	NA	NA
**1.03 (0.41, 1.70)**	−1.83 (−3.79, 0.14)	−0.85 (−1.83, 0.19)	0.09 (−1.17, 1.41)	−1.64 (−3.55, 0.28)	0.53 (−1.33, 2.45)	−0.02 (−2.04, 2.00)	0.19 (−1.42, 1.82)	***L. plantarum***	0.36 (−1.2, 1.9)	NA	−1.1 (−2.5, 0.35)
0.23 (−1.11, 1.55)	**−2.64 (−4.94, −0.38)**	**−1.66 (−3.04, −0.28)**	−0.71 (−2.45, 1.02)	**−2.45 (−4.70, −0.23)**	−0.27 (−2.49, 1.94)	−0.83 (−3.17, 1.48)	−0.62 (−2.63, 1.37)	−0.80 (−2.16, 0.50)	***L. crispatus***	NA	**−1.4 (−2.8, −0.032)**
0.05 (−1.41, 1.52)	**−2.82 (−5.18, −0.46)**	**−1.84 (−3.60, −0.05)**	−0.89 (−2.71, 0.95)	**−2.63 (−4.95, −0.33)**	−0.45 (−2.75, 1.85)	−1.01 (−3.41, 1.39)	−0.80 (−2.89, 1.28)	−0.98 (−2.60, 0.59)	−0.18 (−2.15, 1.81)	**AKK**	NA
**1.67 (0.34, 2.97)**	−1.20 (−3.49, 1.06)	−0.22 (−1.60, 1.15)	0.73 (−1.00, 2.45)	−1.01 (−3.26, 1.20)	1.17 (−1.03, 3.37)	0.61 (−1.73, 2.90)	0.83 (−1.18, 2.79)	0.64 (−0.72, 1.91)	1.44 (−0.07, 2.95)	1.62 (−0.38, 3.57)	***L. fermentum***

The bold value indicate that the difference between the two probiotics is statistically significant.

#### 3.4.2 Colon length

Thirty-four studies reported the CL, including 630 ulcerative colitis models, involving 20 kinds of probiotics. The network evidence diagram is shown in Figure 3. Compared with DSS, 19 kinds of probiotics could improve colon injury, among which are *L. rhamnosus* (MD = −1.22, 95% CrI (−2.21, −0.21)), *T. halophilus* (MD = −2.70, 95% CrI (−4.74, −0.67). −1.05)), *L. plantaru* (MD = −1.73, 95% CrI (−2.27, −1.20)), and *L. fermentam*.

(MD = −2.37, 95% CrI (−3.65, −1.11)) were statistically significant. *L. bulgaricus* (MD = 2.09, 95% CrI (0.13, 4.04)), *L. paracasei* (MD = 2.08, 95% CrI (0.10, 4.04)), *L. reuteri* (MD = 2.88, 95% CrI (0.44, 5.33)), *E. cristatum* (MD = 2.36, 95% CrI (0.29, 4.42)), *B. bifidum* (MD = 2.13, 95% CrI (0.21, 4.07)), *S. thermophilus* (MD = 2.50, 95% CrI (0.08, 4.91)), *T. halophilus* (MD = 4.03, 95% CrI (1.37, 6.70)), *E. faecium* (MD = 4.09, 95% CrI (1.68, 6.49)), *S. boulardii* (MD = 2.64, 95% CrI (0.18, 5.09)), *L. plantarum* (MD = 3.07, 95% CrI (1.37, 4.77)), and AKK (MD = −2.72, 95% CrI (−5.44, −0.01)), and *L. fermentum* (MD = −3.70, 95% CrI (−5.58,−1.84)) was obviously superior to *L. crispatus* and *E. faecium* is obviously superior to *L. paracasei* (MD = −2.02, 95% CrI (−3.99, −0.04)), LB-9 (MD = −2.50, 95% CrI (−4.89, −0.11)), and *B. bifidum* (MD = −1.96, 95% CrI (−3.89,−0.02)). In addition, *L. fermentum* was significantly better than *L. bulgaricus* (MD = −1.61, 95% CrI (−3.03, −0.22)), *L. paracasei* (MD = −1.63, 95% CrI (−3.25, −0.03)), *B. bifidum* (MD = −1.57, 95% CrI (−3.14,−0.01)), and *L. crispatus* (MD = -3.70, 95% CrI (−5.58, −1.84)). The specific data are shown in Supplementary Table S3. According to the SUCRA probability results (Figure 4), *E. faecium* (89.9%)>*T. halophilus* (87.16%)>*L. fermentum* (85.88%)>*L. plantarum* (73.45%)>*L. reuteri* (63.66%)>AKK (58.13%)>*S. boulardii* (56.68%)>*L. rhamnosus* (54.9%)>*S. thermophilus* (52.53%)>*E. cristatum* (48.27%)>*L. kefiranofaciens* M1 (44%)>*C. butyricum* (43.44%)>*L. brevis* (42.86%)>*L. casei* (41.16%)>*B. bifidum* (40.02%) > *L. bulgaricus* (38.64%) > *L. paracasei* (38.03%) > LB-9 (25.62%) > DSS (13.22%) > *L. crispatus* (48.27%), suggesting that *E. faecium* was the best strain to alleviate the colon injury of UC.

#### 3.4.3 Disease activity index

Twenty-five studies reported DAI, a total of 472 UC models, involving 17 probiotics. The network evidence diagram is shown in Figure 3. The DAI of seven probiotics was better than that of DSS, among which *L. reuteri* (MD = 6.18, 95% CrI (2.08, 10.22)), *L. acidophilus* (MD = 2.97, 95% CrI (0.15, 5.83)), *B. bifidum* (MD = 2.87, 95% CrI (0.91, 4.95)), *L. plantarum* (MD = 2.53, 95% CrI (1.08, 3.95)), and AKK (MD = 4.96, 95% CrI (0.04, 9.87)) had statistical significance. Specific data are shown in Supplementary Table S4. According to the SUCRA probability results (Figure 4), *L. reuteri* (90.18%)>AKK(78.45%)>*L. paracasei* (71.39%)>*L. rhamnosus* (69.5%)> *E. durans* (61.46%)>*L. acidophilus* (58.73%)>*B. bifidum* (57.84%)>*E. cristatum* (55.96%) >*L. plantarum* (51.9%)>*L. bulgaricus* (49.89%)>*T. halophilus* (44.43%)>*L. fermentum* (40.67%)>*C. butyricum* (35.78%)> *S. thermophilus* (34.08%)>*L. casei* (31.96%) > *L. crispatus* (28.15%) > LB-9 (27.47%) > DSS (12.18%), suggesting that *L. reuteri* was the best strain to reduce the DAI index of UC.

#### 3.4.4 Historical score

Fifteen studies reported HIS, a total of 246 UC models, involving 10 kinds of probiotics. The network evidence diagram is shown in Figure 3. The HIS of 10 kinds of probiotics was better than that of DSS, among which the HIS of *L. reuteri* (MD = 6.61, 95% CrI (2.00, 11.21)), *L. acidophilus* (MD = 7.56, 95% CrI (4.24, 10.79)), *L. plantarum* (MD = 3.58, 95% CrI (0.95, 6.33)), and *L. acidophilus* was significantly better than that of *L. paracasei* (MD = 5.75, 95% CrI (0.05, 11.29)). 12.73)), *E. durans* (MD = −6.43, 95% CrI (−11.99,−0.77)), and AKK (MD = −5.54, 95% CrI (−10.10,−0.86)). Supplementary Table S5 shows the specific data. According to the SUCRA probability results (Figure 4), *L. acidophilus* (94.42%)>*L. reuteri* (87.87%)>*L. plantarum* (65.1%)>*B. bifidum* (57.5%)>*S. thermophilus* (54.14%) > AKK(43.08%)>*C. butyricum* (42.56%)>*L. paracasei* (40.29%) > *E. durans* (31.04%) > *L. casei* (21.79%) > DSS (12.2%), suggesting that *L. acidophilus* was the best strain to reduce the HIS index of UC.

#### 3.4.5 Tumor necrosis factor-α

Twelve studies reported serum TNF-α, including 279 UC models, involving 11 kinds of probiotics. Figure 3 shows the network evidence diagram. Compared with DSS, 11 kinds of probiotics could reduce the content of TNF-α in serum, among which *L. rhamnosus* (SMD = 5.92, 95% CrI (1.11, 10.73)), *L. plantarum* (SMD = 4.10, 95% CrI (1.47, 6.72)), *L. crustorum* (SMD = 6.42, 95% CrI (1.55, 11.28)), and *L. coryniformis* (SMD = 7.59, 95% CrI (2.58, 12.60)) had a statistically significant difference, *L. bulgaricus* (SMD = −6.38, 95% CrI (−12.45,−0.31)) and AKK (SMD = −7.12, 95% CrI (−14.22,−0.02)) were obviously superior to *L. coryniformis*, and the sspecific data are shown in Supplementary Table S6. According to the SUCRA probability results (Figure 4), *L. coryniformis* (90.4%)>*L. crustorum* (81.5%)>*L. rhamnosus* (77.8)>*E. faecium* (63.5%) > *L. plantarum* (63.1%) > *L. acidophilus* (45.4%) > *L. fermentum* (40.1%) > *B. bifidum* (36.3%) > *C. butyricum* (33.6%)>*L. bulgaricus* (29.8%) > AKK (24.9%)>DSS (13.4%), suggesting that *L. coryniformis* was the best strain to reduce the serum TNF-α content of UC.

#### 3.4.6 Zonula occlusion Ns-1

Five studies reported the expression of colon tight junction protein ZO-1, including 109 UC models, involving eight kinds of probiotics. The network evidence diagram is shown in Figure 3. Compared with DSS, seven probiotics could increase the expression of tight junction protein ZO-1, but the difference was not statistically significant. Supplementary Table S7 shows the specific data. According to the SUCRA probability results (Figure 4), *L. acidophilus* (73.68%)>*B. bifidum* (70.33%)> *L. bulgaricus* (59.94%) > *L. rhamnosus* (49.69%) > *C. butyricum* (48.73%) > *L. plantarum* (46.95%) > *L. coryniformis* (36.93%) > *L. crastorum* (33.21%) > DSS (30.53%), suggesting that *L. acidophilus* was the best strain to increase the tight junction protein ZO-1 in UC.

#### 3.4.7 Shannon

Six studies reported the Shannon index, including 96 ulcerative colitis models, four kinds of probiotics. The network evidence diagram was shown in Figure 3. Compared with DSS, 4 probiotics can increase the Shannon index of UC, but the difference is not statistically significant. The specific data are shown in Supplementary Table S8. According to the probability results of SUCRA (Figure 4), *L. rhamnosus* (93.09%) > *L. plantarum* (49.99%) > *T. halophilus* (45.7%) > *E. durans* (38.07%) > DSS (23.15%), suggesting that *L. rhamnosus* was the best flora to improve the Shannon index of UC.

### 3.5 Publication bias

**FIGURE 5:**
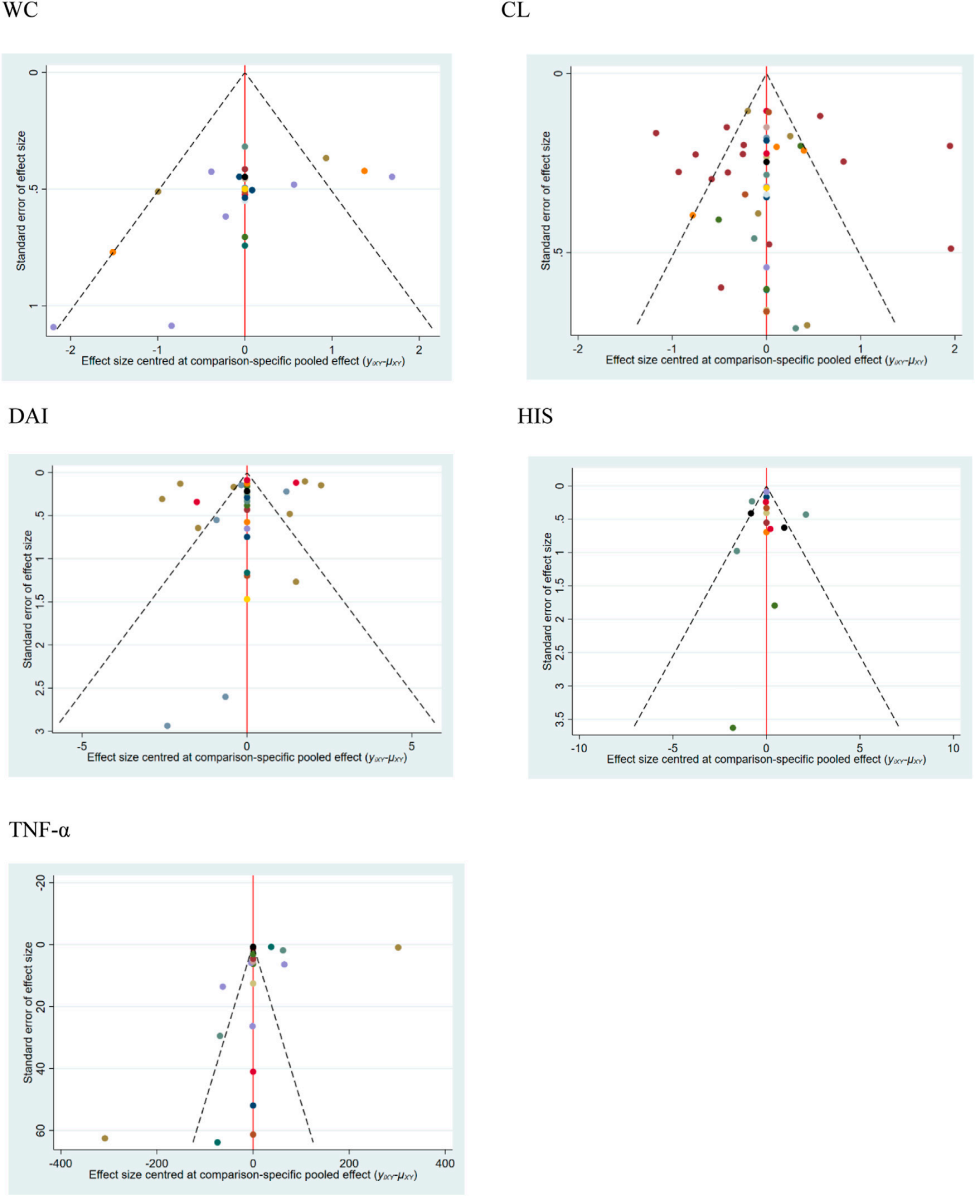
Funnel plots for different outcomes. 1. WC, 2. CL, 3. DAI, 4. HIS, and 5. TNF-α.

The calibration-comparison funnel chart of five outcome indicators (WC, CL, DAI, HIS, and TNF-α) was tested for publication bias, and the results showed that there was asymmetry in scattered points, which indicated that there might be a certain degree of publication bias and small sample size effect in this study. The funnel charts of all the results are shown in Figure 5.

### 3.6 Consistency tests

The inconsistency of WC, CL, DAI, and HIS models was analyzed by the node split method. The results showed that there is no statistical inconsistency, which indicated that the results of the aforementioned outcome indicators are reliable. Supplementary Figure S2 shows the node split analysis diagram.

## 4 Discussion

### 4.1 Research significance

In 2002, probiotics were defined by the World Health Organization as “appropriate intake of living microorganisms that are beneficial to the health of the host” (Wang and Dai, 2020), which can be divided into original bacteria, symbiotic bacteria, and fungi according to the source and mode of action of strains (Zheng et al., 2011). After more than 20 years *in vivo* and *in vitro*, clinical and animal studies had proved the positive effect of probiotics in maintaining the stability of intestinal flora and regulating immunity. Since it is safer than other conventional drugs, it has been widely used in many diseases such as respiratory system, digestive system, and autoimmune diseases, especially inflammatory bowel disease in the gastrointestinal system (Bennet, 2016). At present, a large number of studies have proved that probiotic supplementation can reduce inflammation and induce intestinal environmental balance (Srutkova et al., 2015; Boruel et al., 2002). The most commonly used probiotics include *Lactobacillus*, *Bifidobacterium*, and other probiotics such as Akkermansia muciniphila, *Streptococcus* thermophilus, and *Escherichia coli*.

Ulcerative colitis is a non-specific intestinal inflammatory disease. Studies had shown that probiotics can improve intestinal symptoms, and the treatment of UC aims at maintaining, alleviating, and preventing recurrence (Xue and Huang, 2016). At present, probiotics play a part in UC mainly from the following aspects: 1) Promoting the balance of intestinal flora: The imbalance of intestinal flora is an important reason for the occurrence and development of UC, and probiotics can compete for nutrition and produce antibacterial compounds by occupying adhesion sites on mucosa, thus resisting exogenous pathogens. 2) Maintenance of the intestinal mucosal barrier: A complete intestinal barrier is the basis for maintaining intestinal homeostasis and the most important one is the mechanical barrier. Tight junction proteins are the key components of the mechanical barrier, including occlusal proteins, closing proteins, and junction adhesion molecules*.* Probiotics can protect the intestinal mucosal barrier by regulating tight junction proteins. 3) Regulating immune function: Inflammation is the basis of UC. Probiotics can regulate immune response through a variety of signal pathways, reduce the production of pro-inflammatory cytokines, promote the secretion of anti-inflammatory cytokines, and then, reduce inflammatory response. 4) Enhancing the antioxidant capacity: In the UC state, excessive reactive oxygen species (ROS) can accelerate the inflammatory reaction, leading to cell apoptosis or necrosis, *etc.* Probiotics can reduce the oxidative stress level of colonic mucosa and protect the colonic mucosal barrier (Wang and Dai, 2020). However, not all probiotics have a good curative effect on UC, and the biological effect of probiotics depends on the strain, dose, severity of colitis, and the immune state of the host (Liu et al., 2018), so it is of great significance to seek preclinical evidence for comparing the safety and curative effect of different strains and doses to select probiotics to treat UC in clinic.

### 4.2 Principal finding

In this study, WC, CL, DAI, and HIS are the main outcome indexes because they are the most important and commonly used pathological indexes to evaluate experimental UC in animals. TNF-α is a pro-inflammatory factor to evaluate UC, and ZO-1 is an important protein to evaluate mucosal barrier of UC, and Shannon is an important indicator to reflect the diversity of intestinal flora of ulcerative colitis, so this study regards them as secondary outcome indicators.

This study included 42 studies, including 839 UC models, 24 probiotics. Meta-analysis showed that *L. rhamnosus* had the best effect in relieving weight loss and increasing the Shannon index of UC model. *E. faecium* had the best effect in reducing colon injury; *L. reuteri* had the best effect in reducing DAI; *L. acidophilus* had the best effect in reducing HIS and increasing the expression of tight junction protein ZO-1; and *L. coryniformis* had the best effect in reducing the TNF-α in serum. However, there was no statistical difference between *L. rhamnosus* in increasing the Shannon index and *L. acidophilus* in increasing the expression of tight junction protein ZO-1 compared with the untreated group.

In this study, five outcome indicators were tested for publication bias, and the results showed that there might be some publication bias and small sample size effect in this study. The consistency test of the outcome indicators WC, CL, DAI, and HIS showed that the direct and indirect research of the four outcome indicators is consistent and reliable, but there were lack face-to-face studies in this study.

### 4.3 Preclinical animal models

At present, rodents (rats or mice) are often used in UC model-building (Martín et al., 2017), and the most commonly used induction methods are chemical drugs (Hu et al., 2022), such as dextran sulfate sodium (DSS) and 2,4,6-trinitrobenzene sulfonic acid (TNBS). In this study, the UC model is induced by DSS. Since the UC model induced by DSS has the characteristics of convenient. The animal colitis reaction can be successfully induced by drinking DSS solution freely, and the appropriate drug concentration and administration time can be selected according to the experimental requirements. The specific mechanism is that DSS can destroy animal colon epithelial cells, damage epithelial barrier function, and produce inflammatory substances in intestinal cavity to invade lamina propria and submucosa, and then inducing abnormal immune response in animals (Wirtz et al., 2007). Therefore, the DSS model has advantages in studying the effect of probiotics on UC (Martín et al., 2017). However, the results of network meta-analysis in this study may be limited to the animal model of ulcerative colitis, which is not suitable for use in clinic directly. The difference between the preclinical ulcerative colitis animal model and clinical ulcerative colitis makes our research results need to be treated with caution.

### 4.4 Strengths and limitations of the study

Previous studies and meta-analysis have proved that probiotics have positive effects in improving clinical symptoms, anti-inflammation, and enhancing antioxidant capacity of UC patients (Jia et al., 2018; Kaur et al., 2020; Xie et al., 2022). In this study, the network meta-analysis was used to make preclinical analysis of different strains, which can compare the effects of different strains on ulcerative colitis. Compared with clinical research, it can explore the histopathological effects of probiotics on UC animal models, and provide reference for clinical application of probiotics in the treatment of UC.

In addition, there are still some limitations in this study: 1) The overall quality of the included studies is medium, some studies do not describe the random sequence generation method in detail, and most studies do not mention blind method, distribution concealment, and registration of animal experiments. 2) Some data in this study were obtained indirectly through data extraction tools, which might lead to measurement deviation. 3) By drawing the funnel diagram, it was found that there was a certain degree of publication bias and small sample size effect in this study. 4) In order to improve the research quality, only the English literature was included in this study, which might lead to language bias. 5) The heterogeneity of this study was high, we tried doing the sensitivity analysis and the results were stable. We analyze the heterogeneity might come from animal strains, induction time, disease severity, and so on. 6) The results of this study might only be applicable to preclinical animal models of ulcerative colitis, and the differences between animals and humans make it necessary to be cautious when applying the results of this study in clinic. 7) This study lacks of face-to-face comparison between different probiotics.

## 5 Conclusion

In summary, the results of network meta-analysis showed that different probiotics have different effects on the ulcerative colitis model, and some probiotics have definite effects on improving the histopathological manifestations of UC and reducing inflammatory reaction. However, considering the limitations of this study, preclinical studies that need more large samples and high-quality and more reliable and rigorous experimental designs need to be conducted in the future.

## Data Availability

The original contributions presented in the study are included in the article/Supplementary Material; further inquiries can be directed to the corresponding authors.
