# Supplementary material for: Preclinical evidence of probiotics in ulcerative colitis: a systematic review and network meta-analysis
Source: Front Pharmacol. 2023 Jun 9;14:1187911. doi: 10.3389/fphar.2023.1187911 (PMC10288114; doi:10.3389/fphar.2023.1187911)
Supplement: Supplementary file 1 [file DataSheet1.docx]

Supplementary Table 1: Search strategy

| Database | Search strategies |
| --- | --- |
| Pubmed | ((((((Colitis, Ulcerative[Title/Abstract]) OR (Idiopathic Proctocolitis[Title/Abstract])) OR (Ulcerative Colitis[Title/Abstract])) OR (Colitis Gravis[Title/Abstract])) OR (Inflammatory Bowel Disease, Ulcerative Colitis Type[Title/Abstract])) AND ((Probiotics[Title/Abstract]) OR (Probiotic[Title/Abstract]))) AND ((animals) OR (animal)) |
| Web of science | #5 AND #4 AND #2  5 (ALL=(animals)) OR ALL=(animal)  4 (TS=(Probiotics)) OR TS=(Probiotic)  2 ((((TS=(Colitis, Ulcerative)) OR TS=(Idiopathic Proctocolitis)) OR TS=(Ulcerative Colitis)) OR TS=(Colitis Gravis)) OR TS=(Inflammatory Bowel Disease, Ulcerative Colitis Type) |
| Embase | #14. #6 AND #12 AND #13  #13. #7 OR #8 OR #9  #12. #10 OR #11  #11. 'animals'  #10. 'animal'/exp  #9. 'probiotic':ti,ab  #8. 'probiotics':ti,ab  #7. 'probiotic agent'/exp  #6. #1 OR #2 OR #3 OR #4 OR #5  #5. 'inflammatory bowel disease, ulcerative colitis type':ti,ab  #4. 'colitis gravis':ti,ab  #3. 'idiopathic proctocolitis':ti,ab  #2. 'colitis, ulcerative':ti,ab  #1. 'ulcerative colitis'/exp |

Supplementary Table 2: Summary of risk of bias

| Study | Sequence generation | Baseline characteristics | Allocation concealment | Random housing | Blinding of outcome assessors | Random outcome assessment | Incomplete outcome data | Selective outcome reporting | Other sources of bias |
| --- | --- | --- | --- | --- | --- | --- | --- | --- | --- |
| LEE 2022 | + | + | ? | + | ? | ? | + | + | + |
| Chen 2013 | + | + | ? | + | ? | ? | + | + | + |
| Gao 2021 | + | + | ? | + | + | + | + | + | + |
| Ma 2022 | - | + | ? | + | ? | ? | + | + | + |
| Liu 2016 | + | + | ? | + | ? | ? | + | + | + |
| Chen 2021 | + | + | ? | + | ? | ? | + | + | + |
| Huang 2021 | + | + | ? | + | ? | ? | + | + | + |
| Li 2022 | + | + | ? | + | ? | ? | + | + | + |
| Chae 2018 | ? | + | ? | + | ? | ? | + | + | + |
| Pan 2014 | + | + | ? | + | ? | ? | + | + | + |
| Liu 2011 | + | + | ? | + | ? | ? | + | + | + |
| Din 2020 | + | + | ? | + | ? | ? | + | + | + |
| Shang 2022 | + | + | ? | + | ? | ? | + | + | + |
| Sun 2018 | + | + | ? | + | ? | ? | + | + | + |
| Qin 2022 | + | + | ? | + | ? | ? | + | + | + |
| Kanda 2016 | ? | + | ? | + | ? | ? | + | + | + |
| Qu 2021 | + | + | ? | + | ? | ? | + | + | + |
| Wang 2021 | + | + | ? | + | + | + | + | + | + |
| Cui 2015 | + | + | ? | + | ? | ? | + | + | + |
| Bian 2019 | + | + | ? | + | ? | ? | + | + | + |
| He 2019 | + | + | ? | + | ? | ? | + | + | + |
| Chen 2019 | ? | + | ? | + | ? | ? | + | + | + |
| Hu 2020 | + | + | ? | + | ? | ? | + | + | + |
| Islam 2022 | + | + | ? | + | ? | ? | + | + | + |
| Tong 2021 | + | + | ? | + | ? | ? | + | + | + |
| Yeo 2020 | + | + | ? | + | ? | ? | + | + | + |
| Wan 2022 | + | + | ? | + | ? | ? | + | + | + |
| Dong 2022 | + | + | ? | + | ? | ? | + | + | + |
| Chae 2019 | ? | + | ? | + | ? | ? | + | + | + |
| Zakostelska 2011 | ? | + | ? | + | ? | ? | + | + | + |
| Elian 2015 | ? | + | ? | + | ? | ? | + | + | + |
| SUN 2020 | ? | + | ? | + | ? | ? | + | + | + |
| Choi 2018 | ? | + | ? | + | ? | ? | + | + | + |
| HuangY 2021 | + | + | ? | + | ? | ? | + | + | + |
| Wu 2022 | + | + | ? | + | ? | ? | + | + | + |
| Yu 2020 | + | + | ? | + | ? | ? | + | + | + |
| HuT 2020 | + | + | ? | + | ? | ? | + | + | + |
| Wang 2018 | ? | + | ? | + | ? | ? | + | + | + |
| Khan 2022 | + | + | ? | + | ? | ? | + | + | + |
| Chen 2012 | - | + | ? | + | ? | ? | + | + | + |
| Lu 2022 | + | + | ? | + | ? | ? | + | + | + |
| Lee 2015 | + | + | ? | + | ? | ? | + | + | + |

Supplementary Table 3: league table for CL; Results of the network meta-analysis are presented in the left lower half and results from pairwise meta-analysis in the upper right half In the left lower and upper right half, mean differences lower than 0 favour the row-defining treatment, Bold font indicates significant results. In addition, NA: not available.

| **DSS** | **1.2**  **(0.19, 2.2)** | 1.2  (−0.10, 2.5) | 0.79  (−0.28, 1.8) | 1.5  (−0.25, 3.3) | 0.25  (−1.5, 2.) | 0.79  (−1.0, 2.6) | 0.89  (−0.88, 2.7) | 0.86  (−0.41, 2.1) | 0.81  (−0.11, 1.7) | 1.2  (−0.57, 2.9) | **2.7**  **(0.64, 4.8)** | **2.8**  **(1.0, 4.5)** | 1.3  (−0.50, 3.1) | 0.87  (−0.96, 2.7) | 0.85  (−0.90, 2.6) | **1.7**  **(1.2, 2.3)** | −1.5  (−3.8, 0.87) | 1.4  (−0.76, 3.5) | **2.3**  **(0.31, 4.3)** |
| --- | --- | --- | --- | --- | --- | --- | --- | --- | --- | --- | --- | --- | --- | --- | --- | --- | --- | --- | --- |
| **-1.22**  **(-2.21, -0.21)** | **L.rhamnosus** | NA | NA | NA | NA | NA | NA | NA | NA | NA | NA | NA | NA | NA | NA | NA | NA | NA | NA |
| -0.75  (-1.89, 0.38) | 0.46  (-1.05, 1.97) | **L.bulgaricus** | NA | NA | NA | NA | NA | NA | NA | NA | NA | NA | NA | NA | NA | **2.1**  **(0.30, 3.9)** | NA | NA | 1.4  (−0.41, 3.1) |
| -0.74  (-1.73, 0.25) | 0.47  (-0.93, 1.88) | 0.01  (-1.50, 1.53) | **L.paracasei** | NA | NA | NA | NA | 0.68  (−1.2, 2.6) | NA | NA | NA | NA | NA | NA | NA | NA | NA | NA | NA |
| -1.55  (-3.32, 0.21) | -0.34  (-2.37, 1.68) | -0.80  (-2.89, 1.30) | -0.81  (-2.84, 1.22) | **L.reuteri** | NA | NA | NA | NA | NA | NA | NA | NA | NA | NA | NA | NA | NA | NA | NA |
| -0.25  (-1.94, 1.43) | 0.96  (-1.01, 2.92) | 0.50  (-1.53, 2.54) | 0.49  (-1.46, 2.45) | 1.30  (-1.14, 3.75) | **LB-9** | NA | NA | NA | NA | NA | NA | NA | NA | NA | NA | NA | NA | NA | NA |
| -0.79  (-2.57, 0.99) | 0.42  (-1.62, 2.47) | -0.04  (-2.16, 2.07) | -0.05  (-2.10, 1.99) | 0.76  (-1.76, 3.27) | -0.54  (-3.00, 1.92) | **L.casei** | NA | NA | NA | NA | NA | NA | NA | NA | NA | NA | NA | NA | NA |
| -0.89  (-2.62, 0.83) | 0.33  (-1.67, 2.32) | -0.14  (-2.21, 1.94) | -0.145  (-2.15, 1.85) | 0.66  (-1.80, 3.12) | -0.64  (-3.05, 1.79) | -0.10  (-2.58, 2.37) | **L.kefiranofaciens M1** | NA | NA | NA | NA | NA | NA | NA | NA | NA | NA | NA | NA |
| -1.02  (-2.20, 0.15) | 0.19  (-1.36, 1.73) | -0.27  (-1.90, 1.36) | -0.28  (-1.64, 1.07) | 0.53  (-1.58, 2.65) | -0.77  (-2.82, 1.28) | -0.23  (-2.37, 1.91) | -0.14  (-2.22, 1.96) | **E.cristatum** | NA | NA | NA | NA | NA | NA | NA | NA | NA | NA | NA |
| -0.80  (-1.72, 0.10) | 0.42  (-0.94, 1.76) | -0.05  (-1.50, 1.40) | -0.05  (-1.42, 1.28) | 0.75  (-1.24, 2.73) | -0.55  (-2.48, 1.36) | -0.01  (-2.02, 1.99) | 0.09  (-1.86, 2.03) | 0.23  (-1.26, 1.70) | **B.bifidum** | NA | NA | NA | NA | NA | NA | NA | NA | NA | NA |
| -1.17  (-2.88, 0.55) | 0.05  (-1.93, 2.03) | -0.41  (-2.46, 1.64) | -0.42  (-2.41, 1.55) | 0.39  (-2.08, 2.84) | -0.91  (-3.30, 1.49) | -0.37  (-2.85, 2.11) | -0.27  (-2.71, 2.16) | -0.14  (-2.22, 1.93) | -0.37  (-2.30, 1.59) | **S.thermophilus** | NA | NA | NA | NA | NA | NA | NA | NA | NA |
| **-2.70**  **(-4.74, -0.67)** | -1.48  (-3.75, 0.77) | -1.95  (-4.29, 0.38) | -1.96  (-4.23, 0.30) | -1.15  (-3.83, 1.55) | -2.44  (-5.09, 0.19) | -1.91  (-4.61, 0.78) | -1.81  (-4.49, 0.87) | -1.68  (-4.03, 0.67) | -1.90  (-4.13, 0.34) | -1.54  (-4.20, 1.11) | **T.halophilus** | NA | NA | NA | NA | NA | NA | NA | NA |
| **-2.76**  **(-4.46, -1.05)** | -1.54  (-3.51, 0.43) | -2.00  (-4.05, 0.05) | **-2.02**  **(-3.99, -0.04)** | -1.21  (-3.65, 1.25) | **-2.50**  **(-4.89, -0.11)** | -1.97  (-4.43, 0.50) | -1.87  (-4.29, 0.55) | -1.74  (-3.81, 0.34) | **-1.96**  **(-3.89, -0.02)** | -1.59  (-4.00, 0.82) | -0.06  (-2.71, 2.60) | **E.faecium** | NA | NA | NA | NA | NA | NA | NA |
| -1.31  (-3.09, 0.47) | -0.10  (-2.14, 1.95) | -0.56  (-2.66, 1.56) | -0.57  (-2.61, 1.48) | 0.24  (-2.26, 2.74) | -1.06  (-3.50, 1.40) | -0.52  (-3.03, 2.00) | -0.42  (-2.91, 2.06) | -0.29  (-2.41, 1.84) | -0.51  (-2.51, 1.51) | -0.15  (-2.61, 2.32) | 1.39  (-1.32, 4.10) | 1.45  (-1.02, 3.92) | **S.boulardii** | NA | NA | NA | NA | NA | NA |
| -0.87  (-2.66, 0.93) | 0.35  (-1.70, 2.40) | -0.11  (-2.24, 2.01) | -0.12  (-2.18, 1.92) | 0.69  (-1.84, 3.21) | -0.61  (-3.08, 1.84) | -0.08  (-2.61, 2.45) | 0.02  (-2.47, 2.51) | 0.16  (-1.99, 2.30) | -0.07  (-2.07, 1.95) | 0.30  (-2.19, 2.76) | 1.83  (-0.87, 4.55) | 1.89  (-0.58, 4.36) | 0.45  (-2.10, 2.97) | **C.butyricum** | NA | NA | NA | NA | NA |
| -0.85  (-2.57, 0.87) | 0.37  (-1.63, 2.35) | -0.10  (-2.16, 1.97) | -0.11  (-2.09, 1.88) | 0.70  (-1.76, 3.16) | -0.60  (-3.00, 1.81) | -0.05  (-2.52, 2.40) | 0.04  (-2.40, 2.48) | 0.17  (-1.90, 2.25) | -0.05  (-1.99, 1.89) | 0.32  (-2.12, 2.73) | 1.85  (-0.81, 4.51) | 1.91  (-0.51, 4.31) | 0.46  (-2.02, 2.94) | 0.02  (-2.47, 2.50) | **L.brevis** | NA | NA | NA | NA |
| **-1.73**  **(-2.27, -1.20)** | -0.51  (-1.65, 0.61) | -0.98  (-2.15, 0.19) | -0.99  (-2.12, 0.13) | -0.18  (-2.03 1.66) | -1.48  (-3.24, 0.28) | -0.94  (-2.81, 0.92) | -0.84  (-2.66, 0.97) | -0.71  (-2.00, 0.58) | -0.93  (-1.99, 0.13) | -0.57  (-2.36, 1.21) | 0.97  (-1.14, 3.07) | 1.03  (-0.76, 2.80) | -0.42  (-2.29, 1.43) | -0.86  (-2.74, 1.00) | -0.88  (-2.69, 0.91) | **L.plantarum** | **-2.7**  **(-4.9, -0.57)** | NA | 1.3  (−1.3, 3.8) |
| 1.33  (-0.37, 3.04) | **2.55**  **(0.56, 4.52)** | **2.09**  **(0.13, 4.04)** | **2.08**  **(0.10, 4.04)** | **2.88**  **(0.44, 5.33)** | 1.58  (-0.80, 3.98) | 2.12  (-0.33, 4.58) | 2.22  (-0.22, 4.65) | **2.36**  **(0.29, 4.42)** | **2.13**  **(0.21, 4.07)** | **2.50**  **(0.08, 4.91)** | **4.03**  **(1.37, 6.70)** | **4.09**  **(1.68, 6.49)** | **2.64**  **(0.18, 5.09)** | 2.20  (-0.27, 4.68) | 2.18  (-0.24, 4.60) | **3.07**  **(1.37, 4.77)** | **L.crispatus** | NA | **4.0**  **(1.4, 6.5)** |
| -1.40  (-3.51, 0.71) | -0.18  (-2.51, 2.15) | -0.64  (-3.04, 1.76) | -0.65  (-2.98, 1.68) | 0.15  (-2.61, 2.92) | -1.15  (-3.84, 1.57) | -0.60  (-3.37, 2.17) | -0.51  (-3.23, 2.23) | -0.37  (-2.79, 2.05) | -0.60  (-2.89, 1.71) | -0.23  (-2.94, 2.49) | 1.30  (-1.63, 4.25) | 1.36  (-1.34, 4.08) | -0.08  (-2.85, 2.66) | -0.53  (-3.30, 2.24) | -0.54  (-3.28, 2.17) | 0.34  (-1.85, 2.52) | **-2.72**  **(-5.44, -0.01)** | **AKK** | NA |
| **-2.37**  **(-3.65, -1.11)** | -1.15  (-2.78, 0.46) | **-1.61**  **(-3.03, -0.22)** | **-1.63**  **(-3.25, -0.03)** | -0.82  (-3.00, 1.35) | -2.12  (-4.24, -0.02) | -1.58  (-3.78, 0.61) | -1.48  (-3.65, 0.65) | -1.35  (-3.08, 0.37) | **-1.57**  **(-3.14, -0.01)** | -1.21  (-3.35, 0.92) | 0.33  (-2.08, 2.73) | 0.39  (-1.76, 2.50) | -1.06  (-3.26, 1.14) | -1.51  (-3.71, 0.69) | -1.52  (-3.67, 0.60) | -0.64  (-1.95, 0.66) | **-3.70**  **(-5.58, -1.84)** | -0.97  (-3.46, 1.48) | **L.fermentum** |

Supplementary Table 4: league table for DAI; Results of the network meta-analysis are presented in the left lower half and results from pairwise meta-analysis in the upper right half In the left lower and upper right half, mean differences lower than 0 favour the column-defining treatment, Bold font indicates significant results. In addition, NA: not available.

| **DSS** | −3.9  (−7.9, 0.18) | −1.5  (−5.5, 2.5) | −4.1  (−8.4, 0.19) | **−6.2**  **(−10., −2.1)** | −0.78  (−4.9, 3.3) | −1.1  (−5.3, 3.0) | −2.9  (−7., 1.2) | **−3. 0**  **(−5.8, −0.10)** | −3.3  (−7.5, 0.93) | **−2.9**  **(−5.1, −0.89)** | −1.3  (−5.3, 2.7) | −2.0  (−6.7, 2.6) | −1.4  (−6.0, 3.2) | **−2.5**  **(−4.0, −1.0)** | 0.20  (−3.8, 4.2) | **−5.0**  **(−9.9, −0.06)** | −0.68  (−4.8, 3.4) |
| --- | --- | --- | --- | --- | --- | --- | --- | --- | --- | --- | --- | --- | --- | --- | --- | --- | --- |
| 3.89  (-0.18, 7.95) | **L.rhamnosus** | NA | NA | NA | NA | NA | NA | NA | NA | NA | NA | NA | NA | NA | NA | NA | NA |
| 2.43  (-1.13, 5.97) | -1.46  (-6.84, 3.94) | **L.bulgaricus** | NA | NA | NA | NA | NA | NA | NA | NA | NA | NA | NA | 0.79  (-3.2, 4.8) | NA | NA | NA |
| 4.10  (-0.15, 8.36) | 0.20  (-5.67, 6.10) | 1.67  (-3.86, 7.20) | **L.paracasei** | NA | NA | NA | NA | NA | NA | NA | NA | NA | NA | NA | NA | NA | NA |
| **6.18**  **(2.08, 10.22)** | 2.29  (-3.49, 8.02) | 3.75  (-1.66, 9.13) | 2.08  (-3.85, 7.94) | **L.reuteri** | NA | NA | NA | NA | NA | NA | NA | NA | NA | NA | NA | NA | NA |
| 0.78  (-3.29, 4.86) | -3.11  (-8.84, 2.69) | -1.654  (-7.03, 3.76) | -3.32  (-9.20, 2.57) | -5.40  (-11.15, 0.38) | **LB-9** | NA | NA | NA | NA | NA | NA | NA | NA | NA | NA | NA | NA |
| 1.12  (-3.05, 5.27) | -2.77  (-8.58, 3.04) | -1.31  (-6.79, 4.15) | -2.99  (-8.92, 2.98) | -5.06  (-10.89, 0.78) | 0.34  (-5.49, 6.15) | **L.casei** | NA | NA | NA | NA | NA | NA | NA | NA | NA | NA | NA |
| 2.87  (-1.188, 6.94) | -1.02  (-6.78, 4.73) | 0.43  (-4.92, 5.81) | -1.23  (-7.09, 4.67) | -3.31  (-9.08, 2.44) | 2.09  (-3.68, 7.85) | 1.74  (-4.06, 7.59) | **E.cristatum** | NA | NA | NA | NA | NA | NA | NA | NA | NA | NA |
| **2.97**  **(0.15, 5.83)** | -0.91  (-5.87, 4.05) | 0.55  (-3.99, 5.12) | -1.12  (-6.25, 4.00) | -3.21  (-8.15, 1.81) | 2.19  (-2.78, 7.16) | 1.85  (-3.15, 6.92) | 0.12  (-4.87, 5.08) | **L.acidophilus** | NA | NA | NA | NA | NA | NA | NA | NA | NA |
| 3.28  (-0.94, 7.469) | -0.61  (-6.50, 5.23) | 0.85  (-4.64, 6.35) | -0.81  (-6.81, 5.15) | -2.89  (-8.75, 2.94) | 2.50  (-3.37, 8.36) | 2.16  (-3.75, 8.04) | 0.42  (-5.45, 6.22) | 0.31  (-4.80, 5.38) | **E.durans** | NA | NA | NA | NA | NA | NA | NA | NA |
| **2.87**  **(0.91, 4.95)** | -1.02  (-5.49, 3.61) | 0.44  (-3.54, 4.56) | -1.22  (-5.88, 3.53) | -3.31  (-7.78, 1.32) | 2.09  (-2.40, 6.70) | 1.75  (-2.81, 6.45) | 0.01  (-4.47, 4.60) | -0.11  (-3.53, 3.42) | -0.41  (-5.02, 4.30) | **B.bifidum** | NA | NA | NA | 0.0023  (-4.0, 4.0) | NA | NA | NA |
| 1.30  (-2.76, 5.36) | -2.59  (-8.29, 3.16) | -1.13  (-6.48, 4.24) | -2.80  (-8.65, 3.06) | -4.89  (-10.62, 0.91) | 0.53  (-5.25, 6.31) | 0.18  (-5.65, 6.00) | -1.56  (-7.30, 4.17) | -1.68  (-6.65, 3.27) | -1.98  (-7.80, 3.88) | -1.57  (-6.16, 2.88) | **S.thermophilus** | NA | NA | NA | NA | NA | NA |
| 2.02  (-2.62, 6.66) | -1.87  (-8.03, 4.28) | -0.40  (-6.27, 5.45) | -2.07  (-8.37, 4.22) | -4.15  (-10.31, 2.05) | 1.25  (-4.95, 7.43) | 0.91  (-5.34, 7.13) | -0.83  (-7.03, 5.30) | -0.94  (-6.43, 4.46) | -1.26  (-7.51, 4.98) | -0.85  (-5.97, 4.16) | 0.72  (-5.44, 6.85) | **T.halophilus** | NA | NA | NA | NA | NA |
| 1.36  (-3.25, 5.95) | -2.54  (-8.66, 3.60) | -1.07  (-6.91, 4.70) | -2.73  (-8.99, 3.51) | -4.82  (-10.93, 1.32) | 0.58  (-5.55, 6.72) | 0.24  (-5.98, 6.41) | -1.51  (-7.62, 4.59) | -1.62  (-7.06, 3.77) | -1.92  (-8.14, 4.28) | -1.52  (-6.58, 3.44) | 0.06  (-6.07, 6.16) | -0.66  (-7.20, 5.83) | **C.butyricum** | NA | NA | NA | NA |
| **2.53**  **(1.08, 3.95)** | -1.37  (-5.67, 2.95) | 0.10  (-3.44, 3.65) | -1.57  (-6.07, 2.91) | -3.65  (-7.96, 0.69) | 1.75  (-2.60, 6.04) | 1.41  (-3.01, 5.81) | -0.34  (-4.65, 3.96) | -0.44  (-3.65, 2.74) | -0.75  (-5.18, 3.70) | -0.34  (-2.75, 1.93) | 1.22  (-3.08, 5.53) | 0.50  (-4.35, 5.36) | 1.17  (-3.64, 5.99) | **L.plantarum** | 0.46  (-3.6, 4.5) | NA | -4.2  (-4.5, 3.7) |
| 0.94  (-2.62, 4.49) | -2.95  (-8.34, 2.47) | -1.49  (-6.42, 3.44) | -3.16  (-8.70, 2.38) | -5.24  (-10.62, 0.17) | 0.16  (-5.24, 5.56) | -0.18  (-5.66, 5.33) | -1.92  (-7.31, 3.47) | -2.04  (-6.59, 2.55) | -2.34  (-7.84, 3.18) | -1.92  (-6.06, 2.04) | -0.36  (-5.73, 5.03) | -1.08  (-6.94, 4.75) | -0.42  (-6.20, 5.41) | -1.59  (-5.15, 1.97) | **L.crispatus** | NA | -8.7  (-4.9, 3.2) |
| **4.96**  **(0.04, 9.87)** | 1.08  (-5.33, 7.39) | 2.53  (-3.49, 8.59) | 0.86  (-5.64, 7.37) | -1.21  (-7.59, 5.21) | 4.18  (-2.21, 10.57) | 3.84  (-2.62, 10.30) | 2.10  (-4.30, 8.45) | 1.98  (-3.71, 7.64) | 1.68  (-4.77, 8.17) | 2.083  (-3.26, 7.35) | 3.65  (-2.71, 10.01) | 2.93  (-3.80, 9.70) | 3.60  (-3.10, 10.33) | 2.43  (-2.70, 7.55) | 4.02  (-2.05, 10.10) | **AKK** | NA |
| 1.80  (-1.77, 5.37) | -2.09  (-7.51, 3.30) | -0.62  (-5.55, 4.30) | -2.29  (-7.83, 3.25) | -4.38  (-9.80, 1.04) | 1.02  (-4.40, 6.40) | 0.68  (-4.77, 6.17) | -1.06  (-6.48, 4.35) | -1.17  (-5.74, 3.37) | -1.49  (-6.97, 4.04) | -1.07  (-5.17, 2.92) | 0.50  (-4.90, 5.91) | -0.23  (-6.11, 5.63) | 0.45  (-5.36, 6.25) | -0.73  (-4.30, 2.85) | 0.87  (-3.17, 4.88) | -3.16  (-9.19, 2.91) | **L.fermentum** |

Supplementary Table 5: league table for HIS; Results of the network meta-analysis are presented in the left lower half and results from pairwise meta-analysis in the upper right half In the left lower and upper right half, mean differences lower than 0 favour the column-defining treatment, Bold font indicates significant results. In addition, NA: not available.

| **DSS** | −1.8  (−6.3, 2.7) | **−6.6**  **(−11., −2.0)** | −0.38  (−5., 4.2) | **−7.6**  **( −11., −4.2)** | −1.1  (−5.7, 3.4) | −3.2  (−8.0, 1.4) | −2.9  (−7.5, 1.8) | −2.0  (−6.7, 2.8) | **−3.6**  **(−6.3, −0.94)** | -2.0  (-5.3, 1.3 ) |
| --- | --- | --- | --- | --- | --- | --- | --- | --- | --- | --- |
| 1.82  (-2.70, 6.35) | **L.paracasei** | NA | NA | NA | NA | NA | NA | NA | NA | NA |
| **6.61**  **(2.00, 11.21)** | 4.79  (-1.72, 11.28) | **L.reuteri** | NA | NA | NA | NA | NA | NA | NA | NA |
| 0.39  (-4.14, 4.92) | -1.42  (-7.86, 4.97) | -6.22  (-12.68, 0.26) | **L.casei** | NA | NA | NA | NA | NA | NA | NA |
| **7.56**  **(4.24, 10.79)** | **5.75**  **(0.05, 11.29)** | 0.95  (-4.73, 6.54) | **7.17**  **(1.51, 12.73)** | **L.acidophilus** | NA | NA | NA | NA | NA | NA |
| 1.14  (-3.42, 5.68) | -0.68  (-7.12, 5.72) | -5.47  (-11.97, 0.98) | 0.75  (-5.71, 7.22) | **-6.43**  **(-11.99, -0.77)** | **E.durans** | NA | NA | NA | NA | NA |
| 3.22  (-1.31, 8.00) | 1.40  (-4.91, 8.01) | -3.39  (-9.74, 3.22) | 2.82  (-3.48, 9.44) | -4.34  (-9.82, 1.51) | 2.08  (-4.22, 8.72) | **B.bifidum** | NA | NA | NA | NA |
| 2.86  (-1.78, 7.49) | 1.05  (-5.45, 7.54) | -3.75  (-10.24, 2.79) | 2.48  (-4.05, 8.94) | -4.70  (-10.32, 1.03) | 1.73  (-4.79, 8.26) | -0.34  (-7.09, 6.01) | **S.thermophilus** | NA | NA | NA |
| 1.99  (-2.74, 6.67) | 0.17  (-6.35, 6.69) | -4.63  (-11.21, 1.94) | 1.59  (-4.94, 8.14) | -5.58  (-11.28, 0.20) | 0.85  (-5.70, 7.40) | -1.23  (-7.98, 5.23) | -0.87  (-7.49, 5.72) | **C.butyricum** | NA | NA |
| **3.58**  **(0.95, 6.33)** | 1.76  (-3.50, 7.13) | -3.04  (-8.33, 2.38) | 3.18  (-2.04, 8.55) | -3.98  (-8.12, 0.36) | 2.44  (-2.79, 7.80) | 0.36  (-5.08, 5.64) | 0.71  (-4.60, 6.14) | 1.59  (-3.76, 7.09) | **L.plantarum** | NA |
| 2.02  (-1.23, 5.29) | 0.20  (-5.41, 5.82) | -4.60  (-10.23, 1.05) | 1.63  (-3.97, 7.22) | **-5.54**  **(-10.10, -0.86)** | 0.89  (-4.71, 6.50) | -1.20  (-7.01, 4.35) | -0.85  (-6.48, 4.84) | 0.03  (-5.70, 5.81) | -1.55  (-5.85, 2.60) | **AKK** |

Supplementary Table 6: league table for TNF-α; Results of the network meta-analysis are presented in the left lower half and results from pairwise meta-analysis in the upper right half In the left lower and upper right half, mean differences lower than 0 favour the column-defining treatment, Bold font indicates significant results. In addition, NA: not available.

| **DSS** | **-5.92**  **(-10.73, -1.11)** | **-4.10**  **(-6.72, -1.47)** | -2.00  (-6.69, 2.68) | **-6.42**  **(-11.28, -1.55)** | **-7.59**  **(-12.60, -2.58)** | -1.21  (-4.64, 2.22) | -2.51  (-6.00, 0.98) | -4.24  (-9.34, 0.86) | -1.40  (-6.46, 3.66) | -1.75  (-5.34, 1.83) | -0.48  (-5.51, 4.56) |
| --- | --- | --- | --- | --- | --- | --- | --- | --- | --- | --- | --- |
| **5.92**  **(1.11, 10.73)** | **L.rhamnosus** | -1.82  (-6.45, 2.81) | NA | -0.50  (-5.54, 4.54) | -1.67  (-6.74, 3.39) | NA | NA | NA | NA | NA | NA |
| **4.10**  **(1.47, 6.72)** | -1.82  (-6.45,2.81) | **L.plantarum** | NA | -2.32  (-6.99, 2.34) | -3.50  (-8.25, 1.26) | NA | NA | NA | NA | NA | NA |
| 2.00  (-2.68, 6.69) | -3.91  (-10.63,2.80) | -2.09  (-7.46,3.28) | **L.fermentum** | NA | NA | 0.79  (-3.89, 5.47) | NA | NA | NA | NA | NA |
| **6.42**  **(1.55,11.28)** | 0.50  (-4.54,5.55) | 2.32  (-2.34,6.99) | 4.42  (-2.34,11.17) | **L.crustorum** | NA | NA | NA | NA | NA | NA | NA |
| **7.59**  **(2.58, 12.60)** | 1.67  (-3.39,6.74) | 3.50  (-1.26,8.25) | 5.59  (-1.27,12.44) | 1.17  (-3.88,6.23) | **L.coryniformis** | NA | NA | NA | NA | NA | NA |
| 1.21  (-2.22, 4.64) | -4.70  (-10.61,1.20) | -2.88  (-7.20,1.44) | -0.79  (-5.47,3.89) | -5.21  (-11.16,0.74) | **-6.38**  **(-12.45,-0.31)** | **L.bulgaricus** | -1.29  (-5.41, 2.82) | NA | NA | NA | NA |
| 2.51  (-0.98, 6.00) | -3.41  (-9.34,2.52) | -1.59  (-5.95,2.77) | 0.50  (-5.03,6.04) | -3.91  (-9.89,2.06) | -5.08  (-11.17,1.01) | 1.29  (-2.82,5.41) | **L.acidophilus** | NA | NA | NA | NA |
| 4.24  (-0.86, 9.34) | -1.68  (-8.69,5.33) | 0.14  (-5.60,5.88) | 2.23  (-4.69,9.16) | -2.18  (-9.23,4.87) | -3.35  (-10.50,3.79) | 3.02  (-3.12,9.17) | 1.73  (-4.45,7.91) | **E.faecium** | NA | NA | NA |
| 1.40  (-3.66,6.46) | -4.52  (-11.50,2.46) | -2.70  (-8.40,3.00) | -0.60  (-7.50,6.29) | -5.02  (-12.04,2.00) | -6.19  (-13.31,0.93) | 0.19  (-5.93,6.30) | -1.11  (-7.25,5.04) | -2.84  (-10.02,4.35) | **C.butyricum** | NA | NA |
| 1.75  (-1.83,5.34) | -4.17  (-10.17,1.83) | -2.34  (-6.79,2.10) | -0.25  (-6.15,5.65) | -4.67  (-10.71,1.38) | -5.84  (-12.00,0.32) | 0.54  (-4.42,5.50) | -0.76  (-5.76,4.24) | -2.49  (-8.72,3.75) | 0.35  (-5.85,6.55) | **B.bifidum** | NA |
| 0.48  (-4.56,5.51) | -5.44  (-12.40,1.52) | -3.62  (-9.30,2.06) | -1.53  (-8.40,5.35) | -5.94  (-12.94,1.05) | **-7.12**  **(-14.22,-0.02)** | -0.74  (-6.83,5.35) | -2.03  (-8.16,4.09) | -3.76  (-10.93,3.40) | -0.92  (-8.06,6.21) | -1.28  (-7.45,4.90) | **AKK** |

| **DSS** | 0.24  (−1.1, 1.7) | 0.57  (−1.4, 2.5) | 0.95  (−1.0, 2.9) | 1.7  (−3.0, 6.5) | 0.14  (−1.8, 2.1) | 0.21  (−1.7, 2.2) | 0.30  (−1.6, 2.2) | 0.39  (−1.6, 2.4) |
| --- | --- | --- | --- | --- | --- | --- | --- | --- |
| -0.29  (-1.71, 1.12) | **L.rhamnosus** | NA | NA | NA | −0.45  (−2.4, 1.5) | −0.38  (−2.3, 1.6) | NA | −0.19  (−2.2, 1.8) |
| -0.57  (-2.56, 1.43) | -0.28  (-2.73, 2.17) | **L.bulgaricus** | 0.37  (−3.1, 3.9) | NA | NA | NA | NA | NA |
| -0.95  (-2.98, 1.08) | -0.66  (-3.12, 1.81) | -0.38  (-2.39, 1.64) | **L.acidophilus** | NA | NA | NA | NA | NA |
| -1.71  (-6.49, 3.05) | -1.42  (-6.37, 3.51) | -1.15  (-6.28, 3.96) | -0.76  (-5.89, 4.36) | **B.bifidum** | NA | NA | NA | NA |
| 0.0002  (-1.87, 1.88) | 0.30  (-1.57, 2.16) | 0.57  (-2.16, 3.31) | 0.95  (-1.80, 3.71) | 1.72  (-3.36, 6.81) | **L.crustorum** | 0.069  (−1.9, 2.0) | NA | 0.25  (-1.7, 2.2 ) |
| -0.07  (-1.93, 1.81) | 0.23  (-1.64, 2.10) | 0.50  (-2.23, 3.24) | 0.89  (-1.87, 3.65) | 1.65  (-3.41, 6.74) | -0.07  (-2.05, 1.92) | **L.coryniformis** | NA | 0.18  (-1.8, 2.1) |
| -0.88  (-2.29, 1.69) | -0.007  (-2.44, 2.44) | 0.27  (-2.56, 3.08) | 0.66  (-2.20, 3.48) | 1.41  (-3.70, 6.54) | -0.30  (-3.03, 2.43) | -0.23  (-2.97, 2.50) | **C.butyricum** | NA |
| -0.25  (-2.11, 1.62) | 0.05  (-1.82, 1.92) | 0.32  (-2.41, 3.05) | 0.71  (-2.05, 3.46) | 1.46  (-3.61, 6.57) | -0.25  (-2.23, 1.74) | -0.18  (-2.19, 1.81) | 0.05  (-2.69, 2.78) | **L.plantarum** |

Supplementary Table 6: league table for ZO-1; Results of the network meta-analysis are presented in the left lower half and results from pairwise meta-analysis in the upper right half In the left lower and upper right half, mean differences lower than 0 favour the row-defining treatment, Bold font indicates significant results. In addition, NA: not available.

Supplementary Table 6: league table for Shannon; Results of the network meta-analysis are presented in the left lower half and results from pairwise meta-analysis in the upper right half In the left lower and upper right half, mean differences lower than 0 favour the row-defining treatment, Bold font indicates significant results. In addition, NA: not available.

| **DSS** | 2.7  (−0.22, 5.6) | 0.25  (−2.6, 3.1) | 0.46  (−2.3, 3.3) | 0.52  (−1.1, 2.2) |
| --- | --- | --- | --- | --- |
| -2.69  (-5.54, 0.19) | **L.rhamnosus** | NA | NA | NA |
| -0.25  (-3.05, 2.56) | 2.44  (-1.57, 6.44) | **E.durans** | NA | NA |
| -0.46  (-3.26, 2.32) | 2.23  (-1.81, 6.19) | -0.21  (-4.19, 3.74) | **T.halophilus** | NA |
| -0.52  (-2.15, 1.09) | 2.18  (-1.15, 5.45) | -0.27  (-3.50, 2.96) | -0.06  (-3.28, 3.17) | **L.plantarum** |

Supplementary Figure 1: Convergence diagnosis graph under Bayesian framework for different outcomes

WC


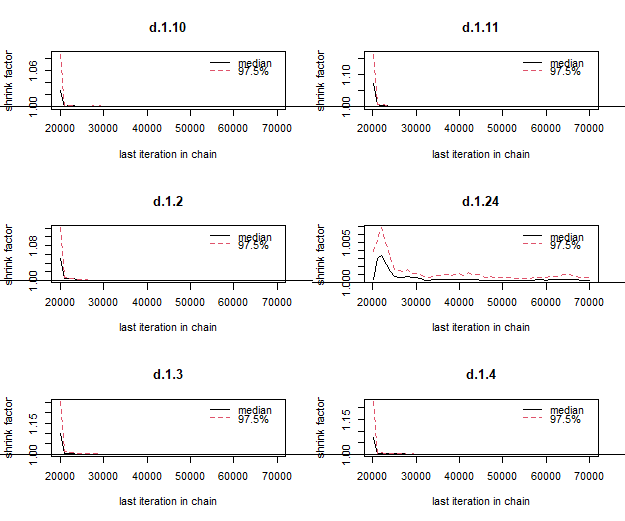

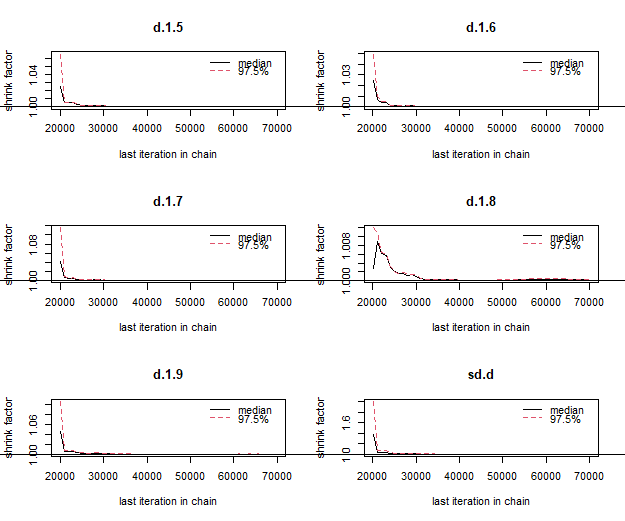


CL


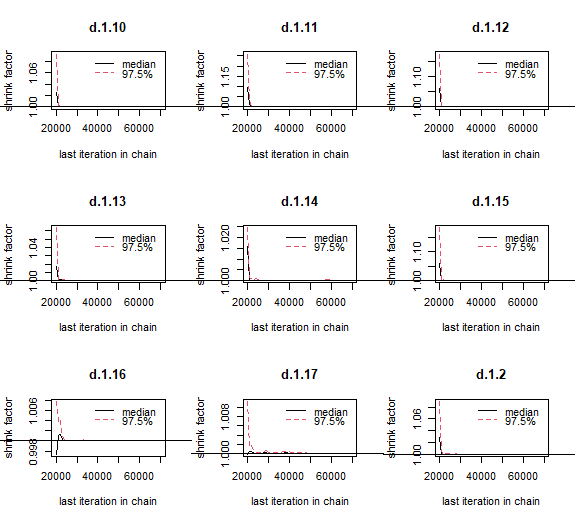

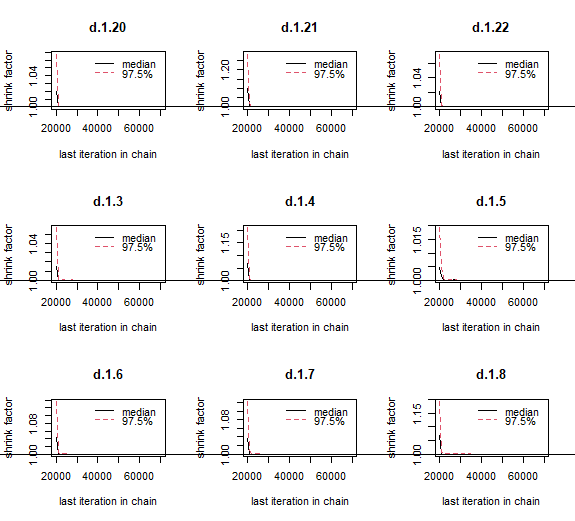


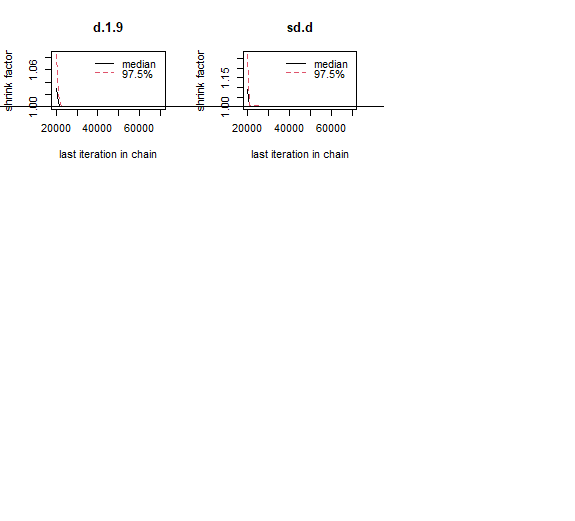


DAI


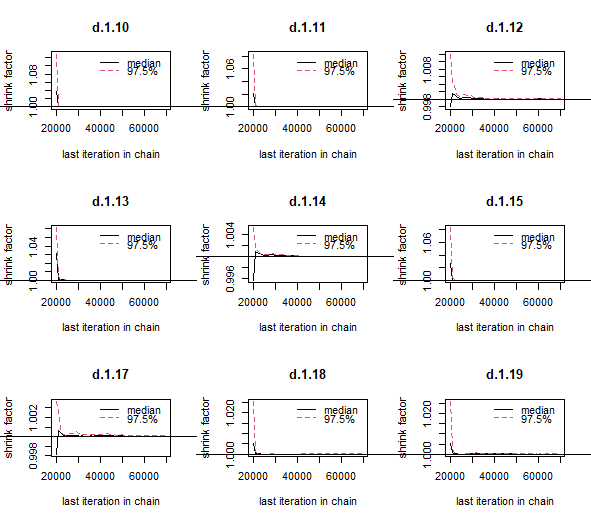

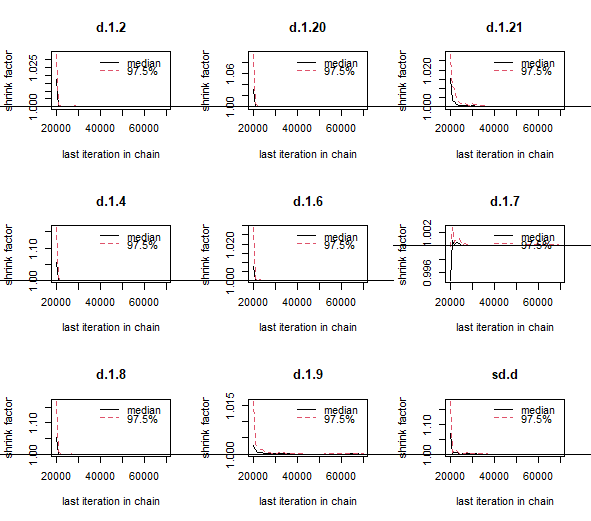


HIS


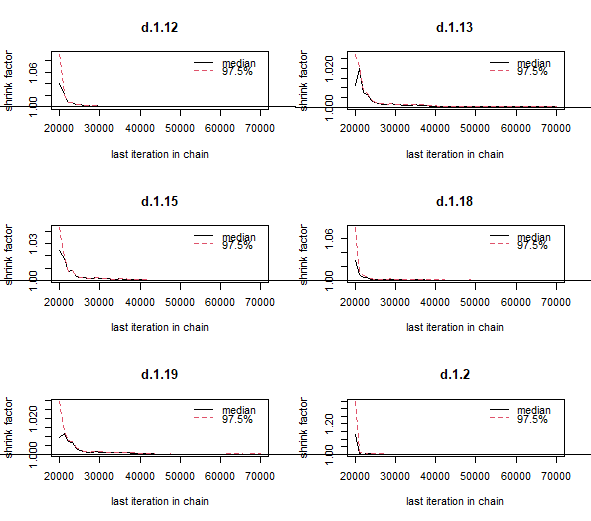

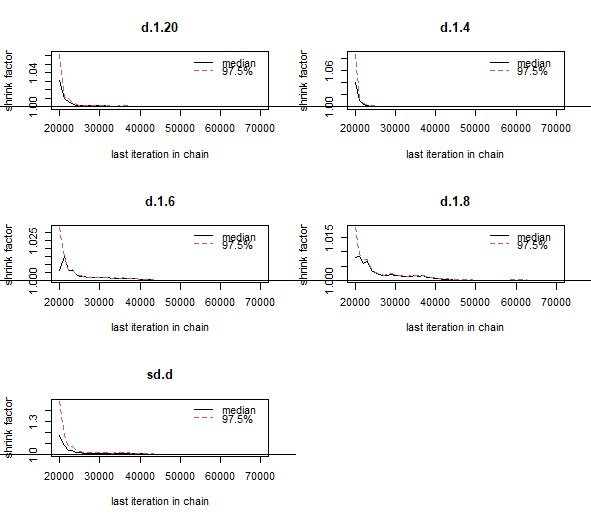


TNF-α


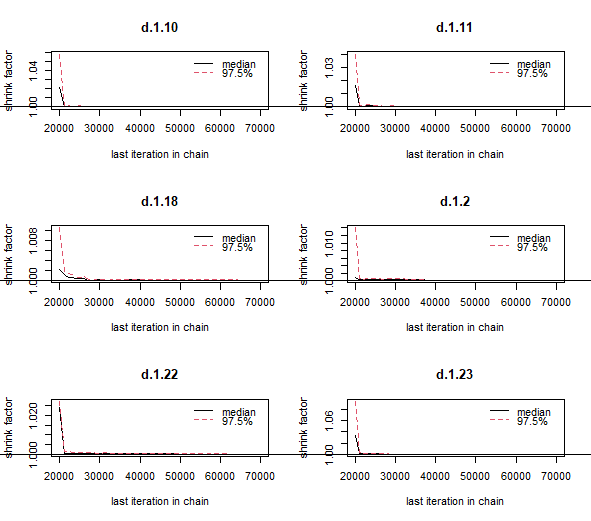

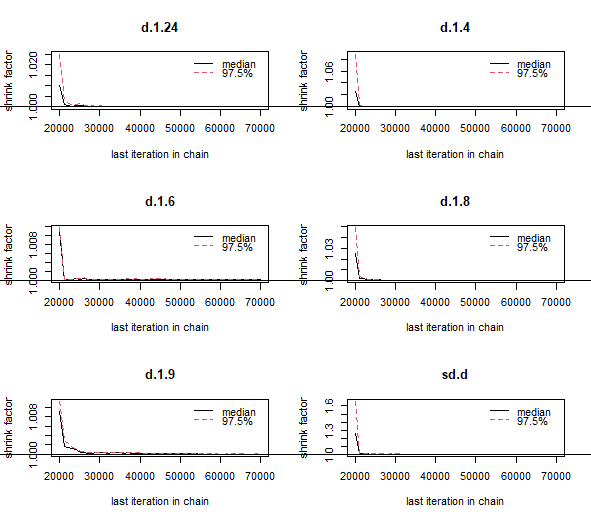


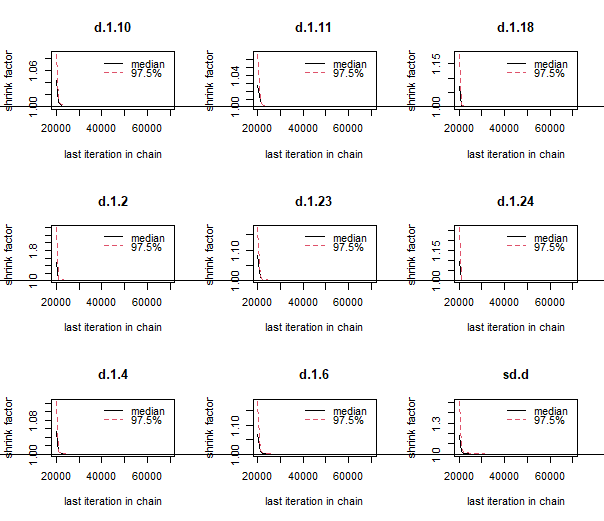
ZO-1


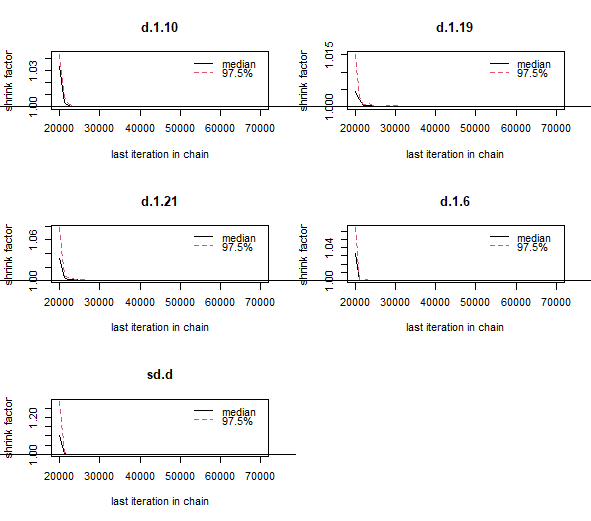
Shannon

Supplementary Figure 2: Nodesplit diagram for different outcomes

WC


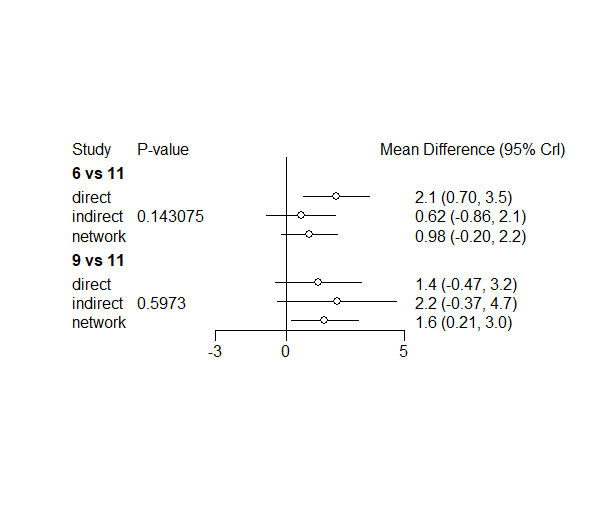

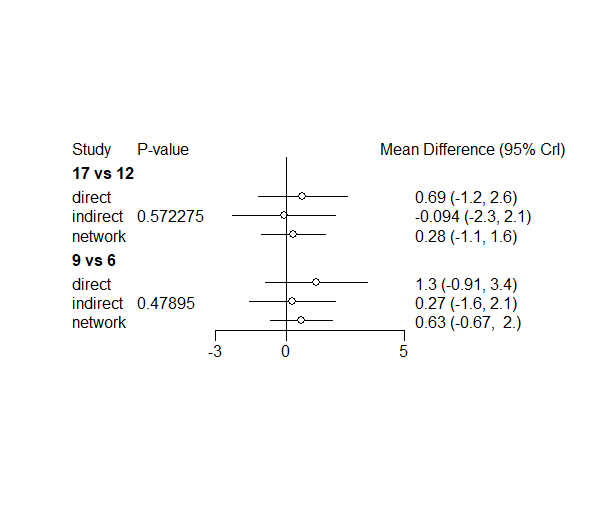


CL


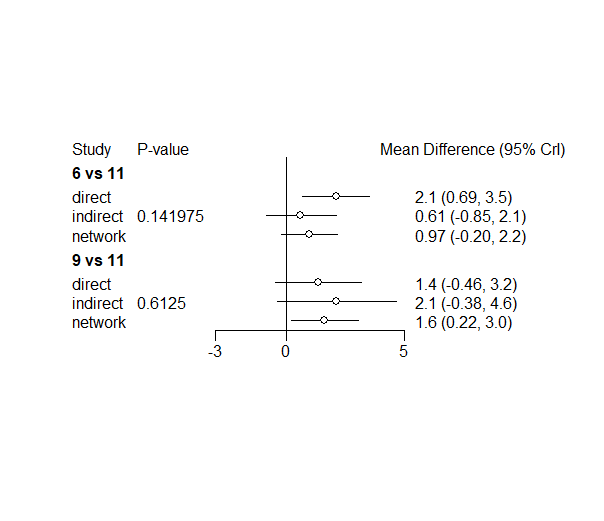

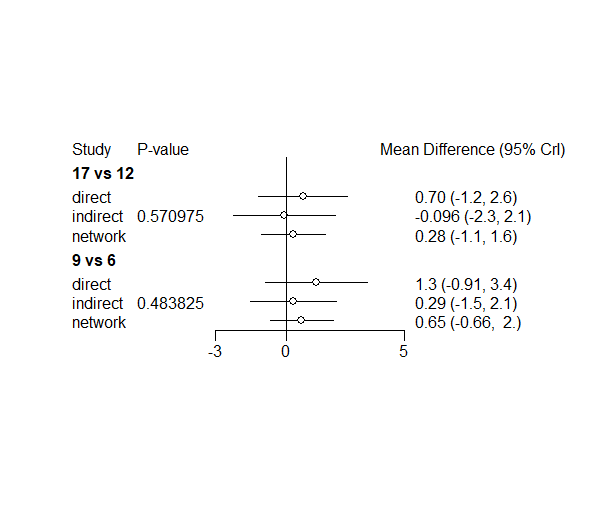


DAI


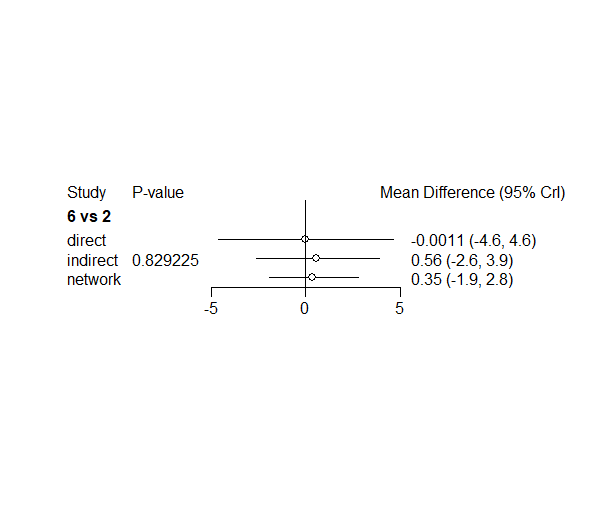


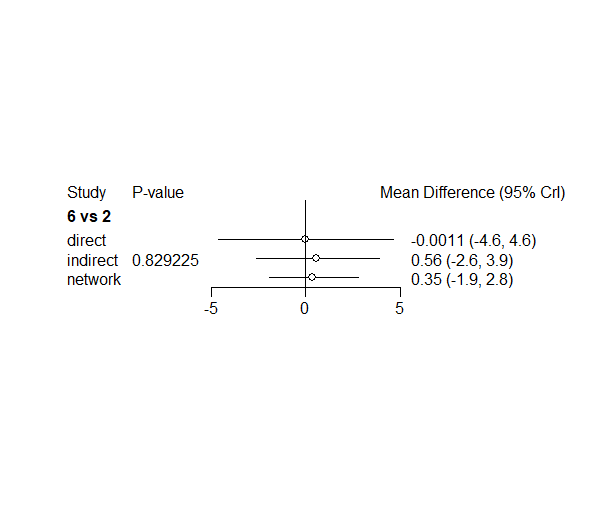
HIS
